# Novel NK1R-Targeted ^68^Ga-/^177^Lu-Radioconjugates with Potential Application against Glioblastoma Multiforme: Preliminary Exploration of Structure–Activity Relationships

**DOI:** 10.3390/ijms23031214

**Published:** 2022-01-21

**Authors:** Joanna Matalińska, Katarzyna Kosińska, Paweł K. Halik, Przemysław Koźmiński, Piotr F. J. Lipiński, Ewa Gniazdowska, Aleksandra Misicka

**Affiliations:** 1 Department of Neuropeptides, Mossakowski Medical Research Institute Polish Academy of Sciences, 5 Pawińskiego Street, 02-106 Warsaw, Poland; jmatalinska@imdik.pan.pl (J.M.); kkosinska@imdik.pan.pl (K.K.); 2 Centre of Radiochemistry and Nuclear Chemistry, Institute of Nuclear Chemistry and Technology, 16 Dorodna Street, 03-195 Warsaw, Poland; p.halik@ichtj.waw.pl (P.K.H.); p.kozminski@ichtj.waw.pl (P.K.); e.gniazdowska@ichtj.waw.pl (E.G.); 3 Faculty of Chemistry, University of Warsaw, 1 Pasteura Street, 02-093 Warsaw, Poland

**Keywords:** L732,138, radiopharmaceuticals, neurokinin-1 receptor antagonist, radioconjugates, targeted radionuclide therapy, glioblastoma multiforme, molecular dynamics

## Abstract

Locoregionally administered, NK1 receptor (NK1R) targeted radionuclide therapy is a promising strategy for the treatment of glioblastoma multiforme. So far, the radiopharmaceuticals used in this approach have been based on the endogenous agonist of NK1R, Substance P or on its close analogues. Herein, we used a well-known, small molecular NK1R antagonist, L732,138, as the basis for the radiopharmaceutical vector. First, 14 analogues of this compound were evaluated to check whether extending the parent structure with linkers of different lengths would not deteriorate the NK1R binding. The tested analogues had affinity similar to or better than the parent compound, and none of the linkers had a negative impact on the binding. Next, five DOTA conjugates were synthesized and used for labelling with ^68^Ga and ^177^Lu. The obtained radioconjugates turned out to be fairly lipophilic but showed rather limited stability in human plasma. Evaluation of the receptor affinity of the (radio)conjugates showed that neither the chelator nor the metal negatively impacts the NK1R binding. The ^177^Lu-radioconjugates exhibited the binding characteristics towards NK1R similar or better than that of the ^177^Lu-labelled derivative of Substance P, which is in current clinical use. The experimental results presented herein, along with their structural rationalization provided by modelling, give insight for the further molecular design of small molecular NK1R-targeting vectors.

## 1. Introduction

Glioblastoma multiforme (GBM, IV WHO grade glioma) is among the most common primary malignant brain tumours [1]. It is also the most aggressive one of all gliomas, with very poor prognosis. The majority of patients do not survive beyond a year, and the median survival time is less than 16 months [2]. The current standard treatments (surgical resection followed by radio- and chemotherapy) are disappointingly ineffective. In almost all patients, a quick GBM recurrence is observed. The problem with treating GBM is in part associated with the heterogenous character of this tumour, its very high proliferation and its infiltrative nature [1]. Another problem is the limited distribution of the systemic drugs (due to the blood–brain barrier) as well as the tumour cells’ resistance to them.

An experimental therapeutic option that holds promise for enhancing the effectiveness of GBM treatment is the locoregionally administered targeted radionuclide therapy (TRT) [3]. The principle of TRT is to deliver α- or β-emitting radionuclides into (or in the close vicinity of) the cancer cells. This is accomplished by the means of radioconjugates of general structure: vector-linker-chelator-radionuclide. The vector fragment (small molecule, peptide or antibody) must have high affinity for a certain molecular target (a receptor) overexpressed at the surface of cancer cells. The linker enables attaching the chelating moiety onto the vector structure without compromising the affinity. A well-tuned radioconjugate diffuses within the tumour and penetrates the infiltrative zone, selectively concentrating the radioactivity around the neoplastic cells and destroying them. At the same time, the locoregional administration (that is into the tumour or into the post-operative resection cavity) allows for bypassing the blood–brain barrier and for sparing the radiation-sensitive organs.

A molecular target particularly suitable for TRT of GBM is the neurokinin-1 receptor (NK1R) [4], since it was shown to be widely overexpressed in GBM as well as in many grade II and grade III gliomas [5]. Moreover, in the normal CNS tissue, NK1R is not highly expressed, except for in a few specific areas (e.g., in the spinal cord) [5]. This should ensure that the critical brain regions are not damaged by the radioactivity.

Targeting NK1R with locally administered radioconjugates was evaluated clinically, showing indeed very promising therapeutic outcomes with little toxicity [5,6,7,8,9]. The attempts that have advanced to clinic so far utilized peptide vectors based on the endogenous NK1R ligand, Substance P (SP) or its close analogues modified to improve in vivo stability [10]. These peptides, conjugated with the macrocyclic chelators (DOTA or DOTAGA), were labelled with ^90^Y, ^177^Lu or ^213^Bi. SP-based radioconjugates labelled with ^225^Ac have also been subject to in vitro [11] and in vivo [8,12] investigations.

Despite the first success of the NK1R-directed TRT concept, the field is open for further developments. For example, it was postulated [8,13] that vectors of lower molecular mass and higher lipophilicity could be more advantageous than SP. Such carriers should distribute more rapidly and more thoroughly, in particular into the walls of the post-surgical cavity. This is especially important in the case of short-lived radionuclides, such as ^213^Bi. In the desire to address this proposition, a search for vectors smaller than SP was initiated. Majkowska-Pilip et al. investigated short Substance P fragments labelled with ^99m^Tc and ^177^Lu [13]. An SP substructure was also used for labelling with ^211^At by Lyczko et al. [14] Recently, we have synthesized radioconjugates based on the FDA-approved small molecular NK1R antagonist, aprepitant [15].

**Figure 1:**
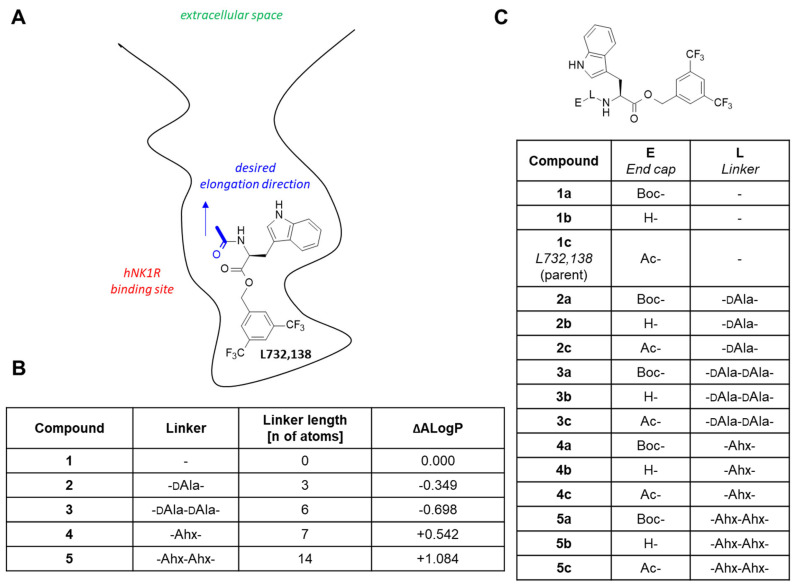
(**A**) Schematic representation of the L732,138 binding model that guided the design. (**B**) The linkers chosen along with their lengths and their predicted effect on lipophilicity expressed as the change of theoretical lipophilicity parameter (ΔALogP). (**C**) The analogues to be synthesized. Abbreviations: Ac—acetyl, Ahx—ε-aminohexanoic acid, Boc—*tert*-butoxycarbonyl, DAla—D-alanine.

In another branch of our research, we have turned our attention to yet other small molecular NK1R antagonist, L732,138 (**1c**, Figure 1C), which had been reported for the first time by MacLeod et al. [16]. Using this compound as a basis for the radiopharmaceutical vector has several advantages from the perspective of structure–activity relationship (SAR) studies. L732,138 exhibits high affinity for NK1R [16]. At the same time, its structure is fairly simple. This makes the generation of analogues required for SAR exploration facile and quick. Moreover, the synthesis of novel vectors from scratch in quantities needed for exhaustive evaluation becomes inexpensive and easy. A potential liability is the presence of the ester linkage, which may be prone to hydrolysis by serum esterases. However, this is not necessarily disqualifying for a compound to be delivered locoregionally, since the activity of the serum enzymes in the cavity or in the cerebrospinal fluid is absent or very low [17]. Note that some of the excellent clinical results obtained so far were obtained with peptide vectors that are susceptible to very quick degradation in plasma [5,7]. Besides, planning the novel conjugates to be used for systemic administration, the issue of stability could be addressed at the later stage by, e.g., isosteric replacements.

Having considered the above-stated points, we designed and prepared several novel analogues of L732,138 to see whether expanding this structure by potential linking elements would not result in a loss of NK1R affinity. In the further step, the novel analogues served for the synthesis of conjugates and radioconjugates, which were subsequently evaluated for physicochemical and biological properties. The results were also analysed in the light of molecular modelling, including docking and molecular dynamics. These efforts are reported in the current contribution.

## 2. Results and Discussion

### 2.1. Structure–Activity Relationships of the Extended L732,138 Analogues

#### 2.1.1. Design Rationale

In building bifunctional conjugates, an essential consideration is how and where to attach the (usually) bulky chelating moiety to the vector fragment so that the receptor affinity is not compromised. Molecular modelling based on homology models of human NK1R (executed before the crystal structures were available) had suggested that L732,138 binds by placing the 3,5-bis(trifluoromethyl)phenyl ring at the very bottom of the binding site (Figure 1A). The *N*-acetyl fragment would be oriented in the direction approximately towards the outlet of the binding site, with the terminal CH_3_-group exposed to the solvent. It had seemed then that expanding the parent molecule by exchanging *N*-acetyl for other *N*-acylating elements should be optimal. Based on the model, we had expected that the direct introduction of a chelator moiety (e.g., DOTA) instead of the *N*-acetyl fragment would not be tolerated due to the bulkiness of the chelator. However, given the uncertainty associated with the homology model of the receptor and the modelling itself, we wanted to probe several depths of chelator placement in the binding site by using linkers of various lengths but not excluding the direct attachment of chelator. We also contemplated the impact of the spacers on the lipophilicity of the compounds (modelled by the change of the theoretical lipophilicity parameter, ALogP). From the point of view of pharmacology, lipophilicity is one of the most important physicochemical parameters for drugs and other medical preparations, because it characterizes their ability to distribute and to accumulate in organisms [18,19].

As a result of these considerations, it was decided to prepare five series of compounds (**1**–**5**) with linkers, as given in Figure 1B. At the ‘end’ of the *N*-terminus, the compounds were to have an unprotected amino group (**b**), *N*-*tert*-butyloxycarbonyl group (**a**) or *N*-acetyl group (**c**) (Figure 1C). Overall, this part of our work was meant to see if introduction of the potential linking fragments would not give loss of NK1R affinity.

#### 2.1.2. Synthesis

**Scheme 1:**
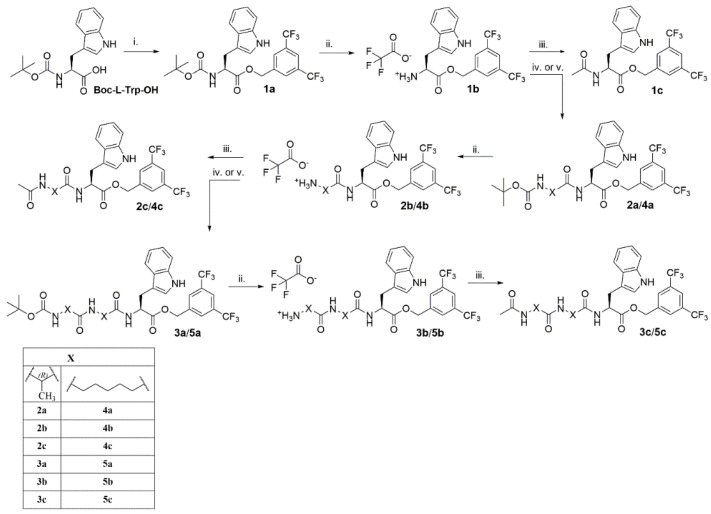
Synthesis of the designed analogues. Reagents and conditions: (i) caesium carbonate (Cs_2_CO_3_), 3,5-bis(trifluoromethyl)benzyl bromide, RT, 18 h, acetonitrile (ACN); (ii) trifluoroacetic acid (TFA): dichloromethane (DCM) (1:1), RT, 1h; (iii) acetic anhydride (Ac_2_O), *N*,*N*,*N′*,*N′*-tetramethylguanidine (TMG), 0 °C–RT, 1,5 h, dimethylformamide (DMF); (iv) 6-(*N-*(*tert*-butyloxycarbonyl)-amino)hexanoic acid *N*-succinimidyl ester (Boc-6-Ahx-OSu), TMG, RT, 6 h, DMF; (v) *N*-hydroxysuccinimide (HOSu), DCC, *N-*(*tert*-butyloxycarbonyl)-D-alanine (Boc-D-Ala-OH), TMG, 0 C- RT, 2 days, DMF.

The synthesis of the designed analogues was accomplished by the divergent approach outlined in Scheme 1. Starting from Boc-L-Trp-OH, the ester **1a** was obtained by *O*-alkylation by the appropriate bromide under standard conditions. Upon deprotection of the Boc group with trifluoroacetic acid (TFA), **1b** was obtained. This compound served either for *N*-acetylation (to give L732,138, **1c**) or for attaching the linking moieties. The latter was carried out by coupling **1b** with the *N*-hydroxysuccinimide (NHS) active esters of the *N^ε^*- or *N^α^*-protected aminohexanoic acid (Ahx; the ester preformed) or D-alanine (DAla; the ester formed in situ). All further analogues were furnished by this sequence of reactions (deprotection followed by acetylation or coupling and so on). In total, 15 compounds were synthesized and isolated, 13 of which are reported for the first time.

#### 2.1.3. Binding Affinity Determination

The prepared analogues were tested as to their affinity for the rat and human NK1 receptors (rNK1R and hNK1R, respectively). This parameter is responsible for the weak or strong binding of tested compounds to the receptor, which results in the quality of the diagnostic imaging and/or the effectiveness of the therapy. Radiopharmaceuticals with high affinity to receptors cause selective destruction of pathological cells and do not affect healthy cells. Testing the compounds at receptors coming from different species is justified by the fact that some NK1R ligands show markedly different affinities for the human variant and the rat variant [20]. While the pursued radioconjugates are intended for potential use in human medicine, much of the in vivo testing is usually carried out in rats; hence, the information on the possible interspecies differences in affinity may be of value. The results of the binding assays are given in Table 1.

The affinities for the human receptor will be discussed first and in detail. The parent L732,138 (**1c**) in our hands exhibits nanomolar affinity for hNK1R with IC_50_ = 25.6 nM. The deacetylated analogue (**1b**) binds 14× weaker (IC_50_ = 358.9 nM), while the Boc-protected derivative (**1a**) is a 7× weaker NK1R ligand (IC_50_ = 180.4 nM) than the parent compound. Similar hNK1R affinity order (*N*-acetyl > *N*-Boc > amino analogue) was reported previously for derivatives with 3,5-dimethyl substitution at the phenyl ring [21]. Vardanyan et al. previously reported the analogue with free amino group **1b** but in the form of ionic pairs with a few fentanyl carboxylate derivatives as the anions for which they found hNK1R binding with Ki in the order of 20–40 nM [22].

Expanding the parent compound by a single DAla unit (**2c**) yields an almost identical hNK1R affinity (IC_50_ = 25.4 nM), which is not affected by *N*-deacetylation (**2b**, IC_50_ = 25.4 nM). On the contrary, the Boc protected derivative **2a** is a clearly weaker ligand (IC_50_ = 135.0 nM).

An additional DAla residue is associated with some modest improvement of the affinity (**3c**, IC_50_ = 13.5 nM) compared to the parent. The Boc protected derivative **3a** has similar binding strength, but the *N*-deacetylated derivative **3b** binds slightly worse (IC_50_ = 80.8 nM) compared both to the parent **1c** or to **3c**.

Insertion of a single Ahx fragment leads to minor worsening of the affinity (**4c**, IC_50_ = 83.1 nM) in comparison to L732,138. Slightly better affinity is found for the Boc protected derivative **4a** (IC_50_ = 60.7 nM), and further improvement is observed with the *N*-deacetylated analogue **4b** (IC_50_ = 35.4 nM).

Among the longest analogues (with double Ahx fragment) is the most potent of the novel compounds, the *N*-Boc analogue **5a**, whose IC_50_ equals 4.5 nM and is about five times better than that of L732,138. The *N*-acetylated analogue (**5c**) is also slightly better than the parent with IC_50_ = 14.3 nM. The amino derivative **5b** exhibits binding similar to that of L732,138 (IC_50_ = 20.4 nM).

Overall, in the case of the human receptor, the affinity variations associated with the presence of linkers and/or of the capping groups are rather small. Most of the analogues show decent hNK1R affinity and some of them are better than the parent L732,138 (**1c**) or another reference hNK1R ligand, aprepitant (in our hands, IC_50_ = 27.7 nM).

Regarding the rat receptor, the parent L732,138 (**1c**) in our hands exhibits micromolar affinity for rNK1R with IC_50_ = 2879 nM. The difference in L732,138 binding to rNK1R and hNK1r had been already reported [23].

**Figure 2:**
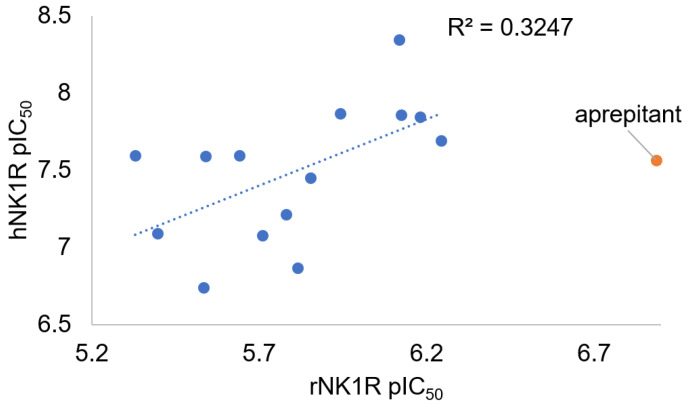
Plot of the hNK1R versus rNK1R binding affinity for compounds **1a**–**5c**. The affinities are shown as the negative logarithm of the IC_50_ value (pIC_50_). Compound **1b** is excluded for having indeterminate rNK1R affinity (IC_50_ > 10 μM). The data for aprepitant are given for reference.

Four novel analogues exhibit submicromolar IC_50_ values (**3a**, **5a**, **5b** and **5c**), and the majority of the remaining ones are slightly better rNK1R ligands than the parent. The trends in structure/affinity changes are not parallel when comparing hNK1R and rNK1R binding. There is only a weak correlation (R^2^ = 0.33) between the human and rat receptor affinities (Figure 2). Importantly, all novel compounds are weaker binders of the rat receptor than Substance P (IC_50_ = 3.6 nM) or aprepitant (IC_50_ = 130 nM [15]).

### 2.2. Radioconjugates

#### 2.2.1. Synthesis of Radioconjugates

**Scheme 2:**
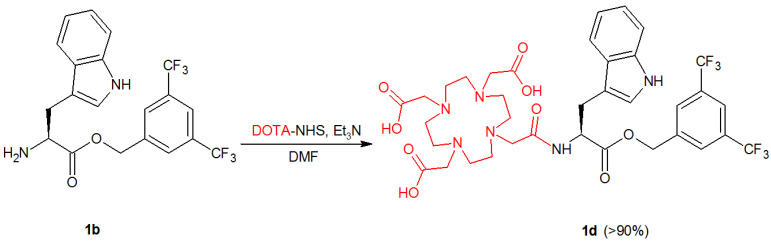
An exemplary synthetic route of conjugation reaction of DOTA-NHS with **1b**. Other *N*-deacetylated derivatives of L-732,138, **2b**–**5b**, follow conjugation reaction in a similar manner.

**Figure 3:**
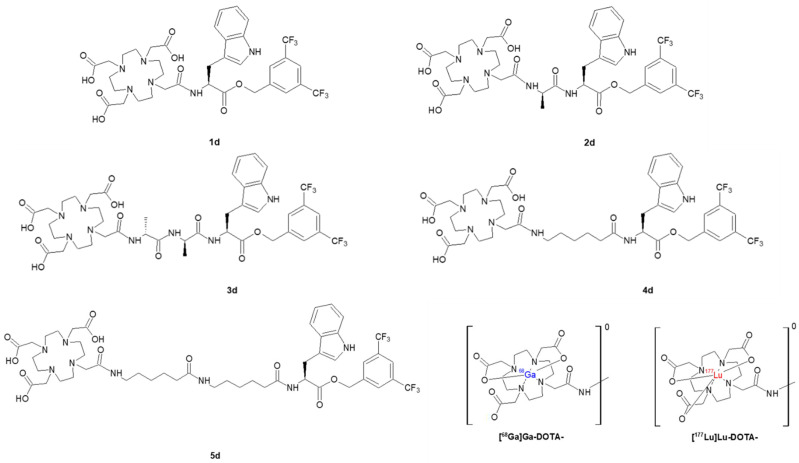
Structures of all DOTA conjugates, **1d**–**5d**, along with [^68^Ga]Ga-DOTA- and [^177^Lu]Lu-DOTA-radiocomplexes present in **[^68^Ga]Ga-1d**–**[^68^Ga]Ga-5d** and **[^177^Lu]Lu-1d**–**[^177^Lu]Lu-5d** radioconjugates.

Since none of the linkers had a profoundly negative or positive effect on hNK1R affinity, it was rational to evaluate radioconjugates based upon each of them. To this aim, the derivatives with the primary amino group (**1b**–**5b**) were reacted with the NHS ester of the cyclic chelator DOTA to obtain the corresponding DOTA conjugates **1d**–**5d** (Scheme 2, Figure 3). The choice of only one radionuclide chelator was dictated by stability issues evaluated previously [15].

**Figure 4:**
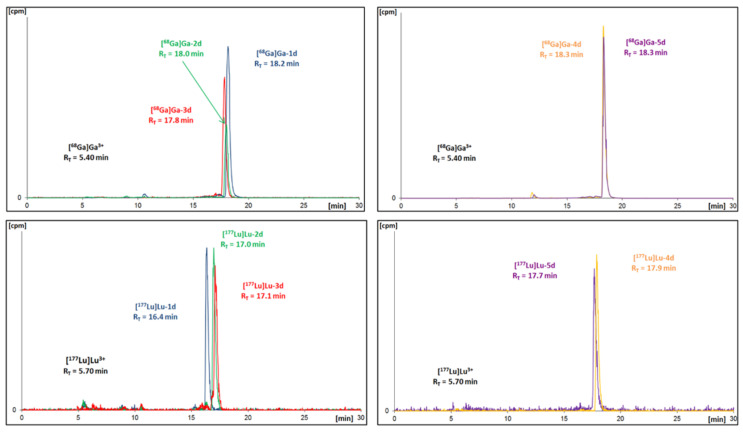
Radiochromatograms of obtained DOTA conjugates radiolabelled with gallium-68 (**upper**) or with lutetium-177 (**bottom**).

Each of the DOTA conjugates was labelled with ^68^Ga or ^177^Lu, providing two series of radioconjugates. Radiolabelling was successfully performed at 95 °C for 10 min at specific activity of around 2 GBq/μmol and in high radiochemical yield above 95%. The obtained radioconjugates were purified using the solid phase extraction (SPE) method before HPLC identification (Figure 4) and further analyses.

In parallel, the verification of the obtained ^68^Ga-radioconjugates was performed by synthesis of the non-radioactive stable gallium reference compounds (**Ga-1d**–**Ga-5d**). These were prepared in an analogous manner, as in the case of ^68^Ga-radioconjugates, followed by the characterization using mass spectrometry. The comparison of HPLC retention times (t_R_) of radioactive and non-radioactive products is presented in Table 2. The values are consistent, corroborating the identity of the conjugates (the differences between them result from the serial connection of UV-Vis and gamma detectors).

#### 2.2.2. Plasma Stability

All obtained radioconjugates, previously isolated from the reaction mixture using the SPE method and being solvent-free, were examined as to their stability in human serum (HS). For this purpose, each radioconjugate was mixed with HS and incubated at 37 °C. At specific time points, small samples of radioconjugate mixture were analysed using the HPLC method for the assessment of the radioconjugate stability.

**Figure 5:**
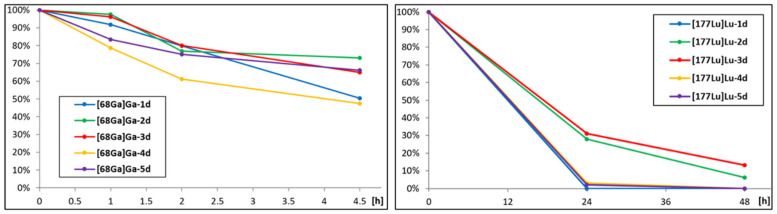
Percentage of intact ^68^Ga-radioconjugates (**left**) and ^177^Lu-radioconjugates (**right**) determined at specific time points during incubation in human serum.

The results of the plasma stability determinations are shown in Figure 5. Exemplary chromatograms are given in the Supplementary Materials in Figures SM-STAB-1 and SM-STAB-2. All the ^68^Ga-radioconjugates begin to decompose as early as at the 1 h time point (79–98% radioactivity remaining). At the 4.5 h time point, 73% of the initial **[^68^Ga]Ga-2d** activity is present in the sample, while only about 50% of the initial **[^68^Ga]Ga-1d** and **[^68^Ga]Ga-4d** is detected.

As to the Lu-labelled radioconjugates, at the 1-day time point, around 30% of **[^177^Lu]Lu-2d** and **[^177^Lu]Lu-3d** remains in the samples. On the other hand, **[^177^Lu]Lu-1d**, **[^177^Lu]Lu-4d**, **[^177^Lu]Lu-5d** are almost undetectable.

We assume that the cause of the gradual decomposition over time may be the presence of an ester moiety in the compound structure. Additionally, in the case of Ahx-containing analogues, which are less stable than the analogues with the DAla moiety, a source of instability may also be the amide bond. Overall, the novel radioconjugates are significantly less stable than the aprepitant-based radioconjugates with DOTA chelator that we reported recently [15].

In parallel, we have corroborated full stability of ^177^Lu-radioconjugates in PBS and cellular medium for 7 days for the purpose of further research.

#### 2.2.3. Lipophilicity Study

A lipophilicity study was also performed right after the SPE method purification and ethanol evaporation. For each DOTA radioconjugate, the lipophilicity value was estimated as the logarithm of the distribution coefficient (D) based on the ratio of the radioactivity of the organic phase to the radioactivity of the aqueous phase in the *n*-octanol/PBS (pH 7.40) system. Simultaneously, the stability of the studied radioconjugate during the experiment was verified through the HPLC analysis of the aqueous phase. LogD_7.4_ values of ^68^Ga- and ^177^Lu-radioconjugates of L-732,138 derivatives are listed in Table 3.

At first glance, it is easily noticed that the ^177^Lu-radioconjugates are more lipophilic by 0.5–0.8 unit than corresponding ^68^Ga-radioconjugates (due to the different complex coordination and presence of the free carboxylate group in the gallium complex). This observation coincides with results for radioconjugates of aprepitant derivatives previously obtained [15]. Moreover, the overall lipophilicity results for radioconjugates of aprepitant and L-732,138 derivatives are fairly similar, despite considerable structure differences between both radioconjugate series. It also shows the substantial hydrophilic impact of DOTA chelator on the resultant lipophilicity of radioconjugates based on small molecular NK1R antagonists.

There is an interesting trend in logD_7.4_ values in relation to the type of the linker used. The most lipophilic radioconjugates are the ones with the Ahx linker (**[^68^Ga]Ga-4d** and **[^177^Lu]Lu-4d**), but surprisingly the least lipophilic are those with a double Ahx linker (**[^68^Ga]Ga-5d** and **[^177^Lu]Lu-5d**). Similarly, the application of only one DAla residue as a linker increased the lipophilicity of ^177^Lu-radioconjugate (**[^177^Lu]Lu-2d**) in comparison to ^177^Lu-radioconjugates based on the L-732,138 derivative without any linker (**[^177^Lu]Lu-1d**), whereas ^68^Ga-radioconjugates with a double DAla linker (**[^68^Ga]Ga-3d**) are characterized by lower lipophilicity to ^68^Ga-radioconjugates without any linker (**[^68^Ga]Ga-1d**). Hypothetically, in the derivatives with doubled linkers, some intramolecular interactions and/or folding occurs, impacting the resultant lipophilicity.

#### 2.2.4. Binding Affinity (Competitive Assay)

The obtained conjugates (both uncomplexed **1d-5d** as well as complexed with cold Ga^3+^, **Ga-1d**–**Ga-5d** series) were evaluated in the competitive binding assay as to their affinity for human and rat NK1R (Table 4).

All conjugates exhibit very good binding to hNK1R (IC_50_ values in the range 4–40 nM). With the exception of the **3c**/**3d** pair, the exchange of the acetyl moiety for DOTA is at least slightly favourable to affinity. The presence of Ga^3+^ cation has little impact on the binding strength. The best hNK1R affinity is found for the longest (**5d**, IC_50_ = 4.2 nM; **Ga-5d**, IC_50_ = 8.7 nM) and the shortest (**1d**, IC_50_ = 7.2 nM; **Ga-1d**, IC_50_ = 5.1 nM) analogues. The latter is surprising in the light of our initial design considerations in which we had predicted that bulky chelator could interfere with the NK1R binding.

#### 2.2.5. Binding Affinity (Saturation Assay)

**Figure 6:**
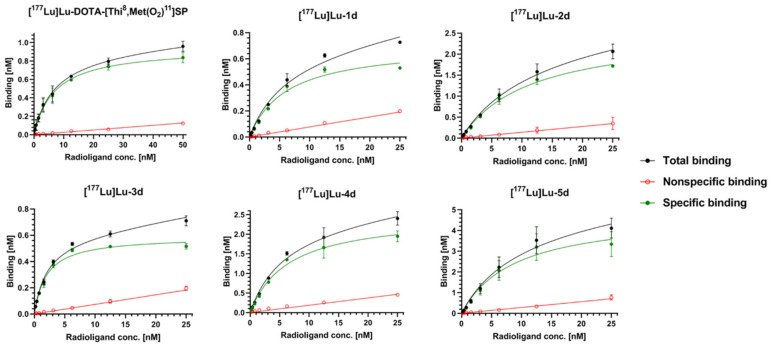
Illustrative binding profiles of ^177^Lu-radioconjugates of Substance P or L-732,138 derivatives.

The analogues labelled with ^177^Lu were assayed for the receptor affinity in saturation binding experiments. For comparative purposes, we have also evaluated a well-known ^177^Lu-radioconjugate of Substance P derivative, i.e., [^177^Lu]Lu-DOTA-[Thi^8^,Met(O_2_)^11^]SP. This very SP derivative is a currently used vector for an α therapy of glioblastoma multiforme [11,24]. The binding parameters are summarized in Table 5, while the illustrative binding profiles are presented in Figure 6.

It is noteworthy that all examined ^177^Lu-radioconjugates exhibit high binding ability towards hNK1R in a quite similar nanomolar range. The highest affinity was obtained for **[^177^Lu]Lu-3d** (K_d_ = 2.002 nM), which was about three times better than the affinity of the reference radioconjugate **[^177^Lu]Lu-DOTA-[Thi^8^,Met(O_2_)^11^]SP** (K_d_ = 6.15 nM). On the other hand, the binding capacity of **[^177^Lu]Lu-3d** was found to be the smallest (B_MAX_ = 0.5939 nM). Better affinity than **[^177^Lu]Lu-DOTA-[Thi^8^,Met(O_2_)^11^]SP** is shown also by **[^177^Lu]Lu-4d** (K_d_ = 5.40 nM), which presents a fairly high binding capacity (B_MAX_ = 2.363 nM), more than 2.5 higher than **[^177^Lu]Lu-DOTA-[Thi^8^,Met(O_2_)^11^]SP** (B_MAX_ = 0.929 nM).

The ^177^Lu-radioconjugate of the derivative without any linker (**[^177^Lu]Lu-1d**) exhibits binding parameters that are a little worse (K_d_ = 6.98 nM, B_MAX_ = 0.762 nM) than the reference radioconjugate of the SP derivative. **[^177^Lu]Lu-2d** and **[^177^Lu]Lu-5d** presented around a 1.5-times worse binding affinity (K_d_ = 10.52 nM and K_d_ = 8.70 nM) than **[^177^Lu]Lu-DOTA-[Thi^8^,Met(O_2_)^11^]SP**, while both radioconjugates show the highest binding capacities (B_MAX_ = 2.497 nM and B_MAX_ = 4.93 nM).

### 2.3. Retrospective Molecular Modelling

#### 2.3.1. Binding Mode of L732,138

In order to rationalize the SAR data found, we performed molecular modelling for some of the reported compounds. The modelling included molecular docking and molecular dynamics (MD) simulations of the ligand–receptor complexes.

**Figure 7:**
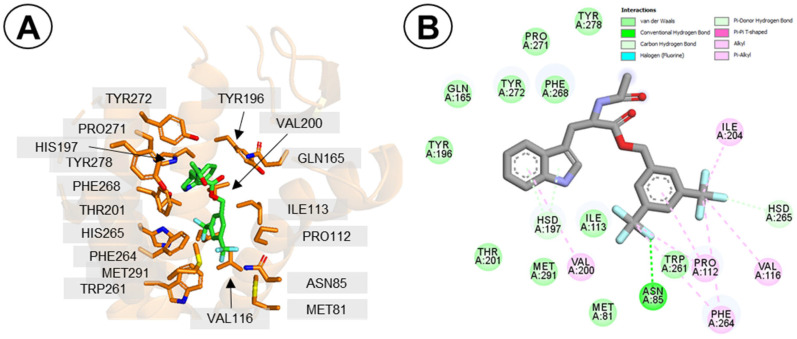
Binding mode of compound **1c** (L732,138) to hNK1R. (**A**) The molecule in the receptor binding site. Only selected residues of the receptor are displayed (orange). The ligand is shown in green. Hydrogen atoms’ display is suppressed. (**B**) Interaction diagram. The interacting residues are coloured according to the legend in the Figure. The presented binding pose is the final snapshot of MD simulation (run nr **1c-1**).

First, we wanted to understand the binding of the parent compound **1c** (L732,138). The compound was docked to 6HLO, 6HLL and 6HLP hNK1R structures [25], and the top scored binding pose was subject to three MD runs of 150 ns production length (RMSD plots Figures SM-MOD-1 to SM-MOD-3). As a result, the binding mode with the following features was obtained (Figure 7). The 3,5-bis(trifluoromethyl)phenyl fragment is located at the bottom of the binding pocket and involved in the hydrophobic interactions with Met81, Asn85, Pro112, Ile113, Val116, Ile204, Trp261, Phe264, His265 and Met291. The indole ring approaches transmembrane helices 6 (TM6) and 7 (TM7). It forms apolar interactions with Gln165, Tyr196, His197 (π-π stacking or π-H-bond donor interactions), Val200, Thr201, Phe268 and Tyr272. The *N*-acetyl fragment of the ligand molecule is exposed to the solvent (in some vicinity of Pro271 and Tyr278 side chains) and directed to the receptor binding site outlet.

Interestingly, the discussed binding pose of **1c** has no direct polar contact between the ligand and the receptor residues. All the interactions are of apolar type. In some of the top docking poses, there was an H-bond between the *N*-acetyl or the ester group and the side-chain of Gln165, but this interaction was not observed during MD runs. Indeed, data from mutagenesis experiments suggest that Gln165 is not important to affinity of L732,138 (**1c**) for hNK1R, as Gln165Ala mutation was associated with a marginal change in affinity (wild type IC_50_ = 3.3 nM; Q165A mutant, IC_50_ = 8.1 nM [26]). The interaction of the indole with H197 is supported by the fact that the His197Ala mutation had been found to be associated with about a ten-fold decrease of L732,138 affinity, while the His197Phe mutation had no effect thereupon [23].

Further support for the modelled binding mode comes from the fact that a partially rigidified analogue with an oxazolidinedione core that was reported to have the slightly lower affinity than the parent L732,138 [27] may be decently overlaid on our proposed binding mode (Figure SM-MOD-4).

In general terms, the premises upon which the design was based (see Section 2.1.1) are corroborated by the modelling with the receptor crystal structures.

#### 2.3.2. Binding Mode of Compound **1d**

Of the experimental results that surprised us during the study were the very good affinities for the conjugates in which DOTA was attached directly to the vector structure, without the linker (**1d**, **Ga-1d** and **[^177^Lu]Lu-1d**). As mentioned in 2.1.1., based on modelling with hNK1R homology models, we had expected **1d** to have worse affinity, for steric reasons. Even now, with the X-ray crystals available, when **1d** was docked to the receptor, the predicted binding poses were significantly displaced compared to ones found for **1c.** In these poses, the 3,5-bis(trifluoromethyl)benzyl fragment was not placed deep in the pocket (Figure SM-MOD-5) but closer to the extracellular space, which could be intuitively expected to give diminished binding strength. This displacement seemed to be driven by the steric requirements and by the bias of docking in favour of polar interactions that could not be found if the ligand’s core remained deep in the pocket.

We suspected then that some receptor flexibility (not captured by docking to a rigid receptor) may enable the formation of favourable contacts with the DOTA moiety and simultaneous accommodation of the core deep in the pocket. To see if it is possible, we built a **1d–hNK1R** complex by manually installing the DOTA fragment into the **1c** structure in the conformation found in the **1c** binding pose from docking and subjected this structure to MD simulations (three runs of 150 ns production length).

**Figure 8:**
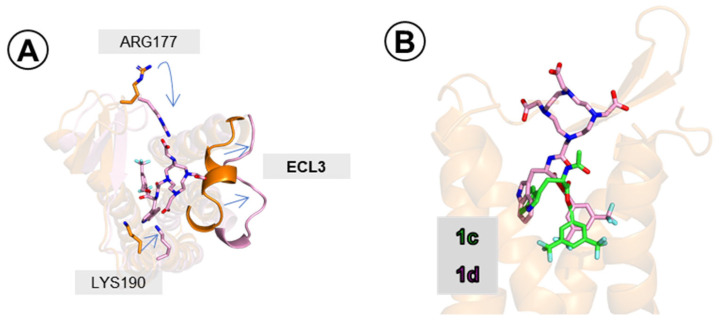
Compound **1d** (pink sticks) in the hNK1 binding site. (**A**) Readjustments of ECL3 and of side-chains of Arg177 and Lys190. In orange are the positions as found in simulation **1c-1**, in pink are the positions as found in simulation **1d-1** (final snapshots). (**B**) Binding pose of **1d** (pink) superposed on that of **1c** (green). Poses taken from final snapshot of **1c-1** and **1d-1** simulations. Only a few receptor (orange) helices are shown.

Comparing simulations with **1c** and **1d**, one can observe some readjustments of the side chains close to the extracellular outlet (Figure 8A) and a change in positioning of the extracellular loop 3 (ECL3). With these, **1d** was able to stay bound deep in the site, with only a slight change of positioning compared to **1c** (with respect to the indole and 3,5-bis(trifluoromethyl)phenyl rings, Figure 8B and Figure SM-MOD-6). A few positively charged or polar side chains (e.g., Arg177, Lys190, Lys194, Gln274; Figures SM-MOD-9 to SM-MOD-11) adopt conformation such that their interactions with DOTA’s carboxylate arms are enabled. These additional interactions formed by the DOTA are likely only slightly beneficial to binding strength due to conformational entropic penalty and/or their solvent exposed positioning. The net result is some minor improvement of affinity.

#### 2.3.3. Binding Modes of Compounds **5a** and **5b**

Another point of interest (with implications for further SAR work) was also the binding mode of the potent long analogues. We studied this problem by the examples of compounds **5a** and **5b**. Docking of these compounds yielded binding poses in which the core fragment is located, as in the case of the parent **1c**, while the linkers are extended and interact with the residues of ECL2 or ECL1. For both compounds, docking finds a set of energetically close binding poses. This picture is somehow corroborated by the MD simulations. Each of the three production runs for **5a** (3 × 210 ns) and **5b** (3 × 250 ns) end with different binding poses (distinct from those found in docking).

**Figure 9:**
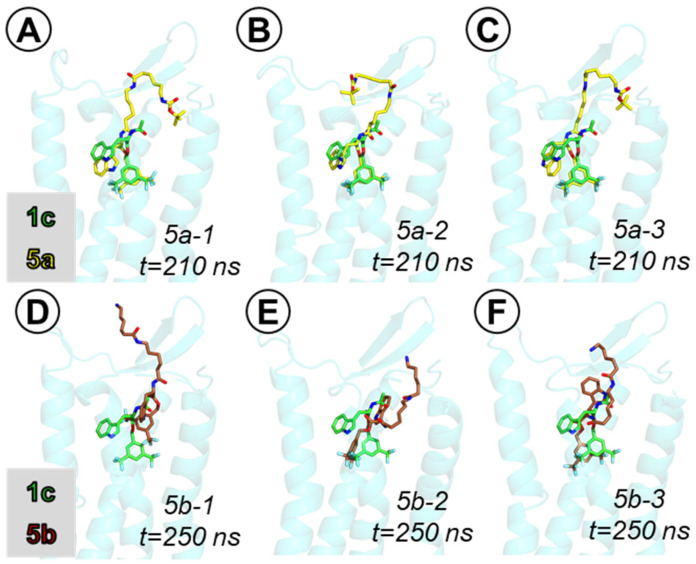
Binding poses of compounds **5a** (yellow sticks, (**A**–**C**)) and **5b** (brown sticks, (**D**–**F**)) in the hNK1 binding site, superposed on the binding pose of compound **1c** (green sticks). Poses taken from final snapshot of the MD simulations. Only a few receptor (light blue) helices are shown.

In the case of the *N*-Boc analogue derivative **5a**, in all three simulations the core fragment remains bound in a manner similar to the parent **1c** (Figure 9A–C, Figures SM-MOD-19 to SM-MOD-21), although some differences can be observed (e.g., formation of a H-bond to Gln165 in two simulations, Figures SM-MOD-19 and SM-MOD-21). The position of the core is rather stable in the simulations (Figures SM-MOD-14 and SM-MOD-15). The long linker arm undergoes significant rearrangement compared to the starting pose (Figure SM-MOD-16), in particular the second Ahx residue changes its position a lot (Figure SM-MOD-17). The final snapshot binding poses (Figure 9A–C) are characterized by a half-coiled conformation of the linker arm, with the *N*-Boc fragment of the molecule locating close to the tips of TM2 or TM6 (possible interaction partners for the *N*-butyloxy group include but are not restricted to the side chains of Tyr92, Tyr278, Gln284; see Figures SM-MOD-19 to SM-MOD-21).

In the case of the amine derivative **5b**, the core fragment has different interactions than the parent compound **1c**. Still, however, the core is bound deep at the similar level as **1c** (in two of three simulations, Figure 9E,F) or retracted slightly higher (in one simulation, Figure 9D). Upon initial rearrangement, the positions of the core fragment (notice that in all three simulations, they are different) remain stable (Figures SM-MOD-23 and SM-MOD-24). On the contrary, the linker arm and especially the Ahx residue with the free terminal amino group retain some mobility (Figures SM-MOD-25 to SM-MOD-27). The linker elements form transient hydrogen bonds and ionic interactions with a few residues, including Tyr92, Asn96, Ile182, Glu183, Glu186, Tyr278, Tyr287, etc. Exemplary snapshots with these contacts are shown in Figures SM-MOD-31 to SM-MOD-33.

## 3. Materials and Methods

### 3.1. Synthesis of L732,138 Analogues

#### 3.1.1. General

All solvents and reagents were obtained in analytical or reagent quality from commercial suppliers and were used without further purification. Thin-layer chromatography (TLC) was used to monitor the progress of the reaction (silica gel 60 F254, Merck, Warsaw, Poland) with UV detection (wavelength: 254 nm) with 1% ninhydrin solution in MeOH as the visualization reagent. Crude products were purified by preparative RP-HPLC on Shimadzu system (Shim-pol, Warsaw, Poland) with reverse phase column Jupiter^®^ 10 µm, 250 × 21.2 mm, Proteo 90 Å, AXIA (Phenomenex, Shim-pol, Warsaw, Poland), using 0.1% TFA in water/acetonitrile (gradients chosen for particular separations) as the mobile phase. The purity of the products was estimated by analytical LC-MS Shimadzu system (Shim-pol, Warsaw, Poland). The conditions of the analytical HPLC method were: gradient of 3–97% phase B in 31 min (phase A: 0.05% aq. formic acid (FA), phase B: ACN + 0.05% FA), total flow 1.2 mL/min, column Jupiter^®^ 4 µm, 250 × 4.6 mm, Proteo 90 Å, UV detection at λ = 210 nm, 254 nm and 280 nm. The identity of the products was confirmed using ESI-MS (Shimadzu LCMS-2020, Kyoto, Japan). The NMR measurements were carried out in DMSO-d6 on Varian-Agilent 500 MHz VNMRS spectrometer at ambient temperature, with trimethylsilane as the internal standard for chemical shifts. ^13^C-NMR spectra of **4b** and **5b** were registered for the hydrochloride salts (given in Supplementary Materials). ^13^C-NMR spectra for **2b**, **2c** and **3c** were not registered.

#### 3.1.2. Synthesis of Compound **1a**

Compound **1a** was prepared according to the procedure described by MacLeod et al. [21] with a change of solvent from dimethylformamide to acetonitrile.

**3,5-bis(trifluoromethyl)benzyl (tert-butoxycarbonyl)-L-tryptophanate**, Boc-L-Trp-O-CH_2_-3,5(CF_3_)_2_Ph, **1a**, white powder, yield 72%, purity > 98% (HPLC-UV), ESI-MS ion found m/z [M+H]^+^: 531.10 calculated [M+H]^+^: 531.17; t_R_ = 21.46 min; ^1^H NMR (600 MHz, DMSO-*d*6) δ (ppm) 10.89 and 10.82 (1H, s), 8.05 and 8.03 (1H, s), 7.97 and 7.95 (2H, s), 7.45 (1H, d, *J* = 7.8 Hz), 7.36 (1H, m), 7.30 (1H, m), 7.14 (1H, bs), 7.03 (1H, m), 6.94 (1H, m), 5.28 (1H, d, *J* = 13.5 Hz), 5.15 (1H, d, *J* = 13.5 Hz), 4.23 (1H, m), 3.12 (1H, m), 3.03 (1H, m), 1.28 and 1.10 (9H, 2s). Some of the signals exhibit doubling that can be ascribed to the presence of two conformers, likely associated with the restricted rotation in the carbamate C–N bond. Similar was found previously for Boc-protected amino acids, e.g., Boc-L-Phe [28]. ^13^C NMR (125 MHz, DMSO-*d*6) δ (ppm), 172.35, 155.93, 140.02, 136.54, 130.72 (q, ^2^*J_CF_* = 32.5 Hz), 128.72 (broad, likely an unresolved quartet with ^3^*J*_CF_ ~4Hz), 127.44, 124.27, 123.72 (q, ^1^*J_CF_* = 270.8 Hz), 122.03 (broad, likely an unresolved quartet with ^3^*J*_CF_ ~4Hz), 121.40, 118.86, 118.32, 111.92, 110.03, 78.80, 64.75, 55.45, 28.44, 27.05.

#### 3.1.3. Synthesis of Compounds **2a** and **3a**

HOSu (1.2 equiv) was added to a solution of Boc-D-Ala-OH (1 equiv) in DMF and the reaction mixture was cooled to 0 °C in an ice bath. Next, DCC (1 equiv) was added, and the mixture was stirred for one hour at 0–5 °C and for further two hours at room temperature. Then, compound **1b** or **2b** (1.2 equiv), respectively, with the addition TMG (1.2 equiv) was added to the reaction mixture. The reaction was allowed to stir at room temperature for 48 h. After this time, the precipitated *N*,*N*′-dicyclohexylurea (DCU) was filtered off and the filtrate was concentrated and extracted with ethyl acetate (AcOEt). The organic phase was dried with anhydrous magnesium sulfate (anh.MgSO4), the solvent was removed in vacuo and the resulting oil was dried.

**3,5-bis(trifluoromethyl)benzyl (tert-butoxycarbonyl)-D-alanyl-L-tryptophanate**, Boc-D-Ala-L-Trp-O-CH_2_-3,5(CF_3_)_2_Ph, **2a**, white powder, yield 38%, purity > 98% (HPLC-UV), ESI-MS ion found m/z [M+H]^+^: 602.15 calculated [M+H]^+^: 602.21; t_R_ = 20.28 min; ^1^H NMR (500 MHz, DMSO-*d*6) δ (ppm), 10.85 (1H, s), 8.22 (1H, d, *J* = 7.5 Hz), 8.05 (1H, s), 7.99 (2H, s), 7.46 (1H, m), 7.31 (1H, m), 7.13 (1H, d, *J* = 2.2 Hz), 7.05 (1H, m), 6.96 (1H, m), 6.72 (1H, d, *J* = 7.3 Hz), 5.29 (1H, d, *J* = 13.6 Hz), 5.20 (1H, d, *J* = 13.6 Hz), 4.56 (1H, m), 4.00 (1H, m), 3.21 (1H, m), 3.10 (1H, m), 1.34 (9H, s), 1.16 and 1.06 (3H, 2d, *J* = 6.9 Hz). ^13^C NMR (125 MHz, DMSO-*d*6) δ (ppm), 173.37, 172.09, 155.33, 139.77, 136.53, 130.64 (q, ^2^*J_CF_* = 32.5 Hz), 128.89 (broad, likely an unresolved quartet with ^3^*J*_CF_ ~4Hz), 127.41, 124.71, 123.67 (q, ^1^*J_CF_* = 271.6 Hz), 121.41, 122.13 (broad, likely an unresolved quartet with ^3^*J*_CF_ ~4Hz), 118.83, 118.27, 111.89, 109.60, 78.45, 64.98, 53.60, 49.90, 28.55, 27.40, 16.68.

**3,5-bis(trifluoromethyl)benzyl (tert-butoxycarbonyl)-D-alanyl-D-alanyl-L-tryptophanate**, Boc-D-Ala-D-Ala-L-Trp-O-CH_2_-3,5(CF_3_)_2_Ph, **3a**, white powder, yield 38%, purity > 98% (HPLC-UV), ESI-MS ion found m/z [M+H]^+^: 673.15 calculated [M+H]^+^: 673.25; t_R_ = 19.60 min. ^1^H NMR (600 MHz, DMSO-*d*6) δ (ppm), 10.83 (1H, s), 8.32 (1H, d, *J* = 7.6 Hz), 8.03 (1H, s), 7.95 (2H, s), 7.70 (1H, d, *J* = 7.3 Hz), 7.43 (1H, m), 7.28 (1H, m), 7.12 (1H, d, *J* = 2.1 Hz), 7.02 (1H, m), 6.92 (1H, m), 6.47 (1H, bs), 5.25 (1H, d, *J* = 13.3 Hz), 5.15 (1H, d, *J* = 13.3 Hz), 4.50 (1H, m), 4.25 (1H, m), 3.90 (1H, m), 3.19 (1H, m), 3.05 (1H, m), 1.32 and 1.27 (9H, 2bs), 1.08 (3H, d, *J* =7.1 Hz), 1.03 (3H, d, *J* = 7.1 Hz). ^13^C NMR (125 MHz, DMSO-*d*6) δ (ppm), 172.72, 172.64, 172.00, 155.60, 139.75, 136.51, 130.74 (q, ^2^*J_CF_* = 32.5 Hz), 128.92 (broad, likely an unresolved quartet with ^3^*J*_CF_ ~4Hz), 127.39, 123.67 (q, ^2^*J_CF_* = 271.3 Hz), 122.17 (broad, likely an unresolved quartet with ^3^*J*_CF_ ~4Hz), 121.40, 124.33, 118.83, 118.25, 111.89, 109.58, 78.59, 65.01, 53.63, 50.17, 48.27, 28.58, 27.34, 18.82, 18.30.

#### 3.1.4. Synthesis of Compounds **4a** and **5a**

Boc-6-Ahx-OSu (1 equiv) was dissolved in the minimum amount of DMF and compound **1b** or **4b** (1.2 equiv) was added, respectively, with the addition of TMG (1.2 equiv). The reaction was carried out for 6 h at room temperature. The reaction mixture was concentrated and extracted with ethyl acetate. The organic phase was dried, the solvent was removed in vacuo and the resulting oil was dried.

**3,5-bis(trifluoromethyl)benzyl (6-((tert-butoxycarbonyl)amino)hexanoyl)-L-tryptophanate**, Boc-Ahx-L-Trp-O-CH_2_-3,5(CF_3_)_2_Ph, **4a**, white powder, yield 57%, purity > 97% (HPLC-UV), ESI-MS ion found m/z [M+H]^+^: 644.15 calculated [M+H]^+^: 644.26; t_R_ = 20.57 min; ^1^H NMR (500 MHz, DMSO-*d*6) δ (ppm), 10.83 (1H, s), 8.32 (1H, d, *J* = 7.4 Hz), 8.04 (1H, s), 7.95 (2H, s), 7.47 (1H, m), 7.32 (1H, m), 7.15 (1H, d, *J* = 2.2 Hz), 7.05 (1H, m), 6.96 (1H, m), 6.69 (1H, bs), 5.28 (1H, d, *J* = 13.5 Hz), 5.17 (1H, d, *J* = 13.5 Hz), 4.53 (1H, m), 3.18 (1H, m), 3.07 (1H, m), 2.82 (2H, m), 2.06 (2H, t, *J* = 7.4 Hz), 1.40 (2H, m), 1.36 (9H, s), 1.29 (2H, m), 1.13 (2H, m). ^13^C NMR (125 MHz, DMSO-*d*6) δ (ppm), 172.89, 172.40, 156.82, 139.96, 136.53, 130.71 (q, ^2^*J_CF_* ~32.5 Hz), 128.66 (broad, likely an unresolved quartet with ^3^*J*_CF_ ~4Hz), 127.45, 124.11, 123.69 (q, ^1^*J_CF_* ~272 Hz), 122.05 (broad, likely an unresolved quartet with ^3^*J*_CF_ ~4Hz), 121.41, 118.83, 118.32, 111.91, 109.91, 77.74, 64.73, 53.85, 35.30, 29.68, 28.70, 27.27, 26.32, 25.26. C_ε_ signal of Ahx overlapped by the DMSO-d6 signal at around 40 ppm.

**3,5-bis(trifluoromethyl)benzyl (6-(6-((tert-butoxycarbonyl)amino)hexanamido)hexanoyl)-L-tryptophanate**, Boc-Ahx-Ahx-L-Trp-O-CH_2_-3,5(CF_3_)_2_Ph, **5a**, white powder, yield 55%, purity > 98% (HPLC-UV), ESI-MS ion found m/z [M+H]^+^: 757.30 calculated [M+H]^+^: 757.34; t_R_ = 19.61 min. ^1^H NMR (500 MHz, DMSO-*d*6) δ (ppm), 10.83 (1H, s), 8.32 (1H, d, *J* = 7.1 Hz), 8.04 (1H, s), 7.96 (2H, s), 7.66 (1H, t, *J* = 5.5 Hz), 7.48 (1H, m), 7.21 (1H, m), 7.15 (1H, d, *J* = 2.1 Hz), 7.05 (1H, m), 6.96 (1H, m), 6.71 (1H, t, *J* = 5.1 Hz), 5.28 (1H, d, *J* = 13.5 Hz), 5.17 (1H, d, *J* = 13.5 Hz), 4.53 (1H, m), 3.19 (1H, m), 3.08 (1H, m), 2.95 (2H, m), 2.87 (2H, m), 2.07 (2H, t, *J* = 7.6 Hz), 2.01 (2H, t, *J* = 7.5 Hz), 1.43 (4H, m), 1.36 (9H, s), 1.33 (4H, m), 1.16 (4H, m). ^13^C NMR (125 MHz, DMSO-*d*6) δ (ppm), 172.88, 172.41, 172.27, 156.01, 139.97, 136.54, 130.72 (q, ^1^*J_CF_* ~32.5 Hz), 128.65 (likely an unresolved quartet with ^3^*J*_CF_ ~4Hz), 127.45, 124.12, 123.68 (q, ^1^*J_CF_* ~272 Hz), 122.05 (likely an unresolved quartet with ^3^*J*_CF_ ~4Hz), 121.41, 118.82, 118.31, 111.91, 109.90, 77.73, 64.73, 55.83, 38.71, 35.84, 35.29, 29.73, 29.38, 28.71, 27.28, 26.45, 25.51, 25.25. C_ε_ signals of both Ahx residues overlapped by the DMSO-d6 signal at around 40 ppm.

#### 3.1.5. Deprotection Reaction (**1b**, **2b**, **3b**, **4b**, **5b**) 

*N^α^*-Boc protected derivatives (**1a** or **2a** or **3a** or **4a** or **5a**) were dissolved in a mixture of TFA and DCM) (1:1). The reaction was left to stir for one hour at room temperature. The reaction mixture was concentrated in vacuo, the product was precipitated using cold diethyl ether and the resulting oil was dried.

**(*S*)-1-((3,5-bis(trifluoromethyl)benzyl)oxy)-3-(1H-indol-3-yl)-1-oxopropan-2-aminium 2,2,2-trifluoroacetate,** TFA*NH_2_-L-Trp-O-CH_2_-3,5(CF_3_)_2_Ph, **1b**, white powder, yield 81%, purity > 98% (HPLC-UV), ESI-MS ion found m/z [M_base_+H]^+^: 431.05, [M_base_−H]^−^: 429.00, [M_sal_t−H]^−^: 542.95 calculated [M_base_+H]^+^: 431.12, [M_base_−H]^−^: 429.10, [M_sal_t−H]^−^: 543.10; t_R_ = 11.38 min; ^1^H NMR (500 MHz, DMSO-*d*6) δ (ppm), 11.03 (1H, s), 8.52 (3H, s), 8.09 (1H, s), 7.99 (2H, s), 7.48 (1H, m), 7.35 (1H, m), 7.22 (1H, d, *J* = 2.2 Hz), 7.07 (1H, m), 6.97 (1H, m), 5.37 (1H, d, *J* = 13.2 Hz), 5.24 (1H, d, *J* = 13.2 Hz), 4.37 (1H, m), 3.30–3.26 (2H, m). ^13^C NMR (125 MHz, DMSO-*d*6) δ (ppm), 169.70, 158.45 (weak, probably q with ^2^*J*_CF_ ~ 30 Hz, consistent with TFA salt), 138.83, 136.66, 130.77 (q, ^2^*J_CF_* = 32.5 Hz), 129.35 (broad, likely an unresolved quartet with ^3^*J*_CF_ ~4Hz), 127.27, 125.29, 123.65 (q, ^1^*J_CF_* = 271 Hz), 122.56 (broad, likely an unresolved quartet with ^3^*J*_CF_ ~4Hz, overlapped with signal for CF_3_ carbon), 121.64, 119.06, 118.26, 117.75 (weak, probably q with ^1^*J*_CF_ ~300 Hz, consistent with TFA salt), 112.74, 106.84, 65.98, 53.27, 26.83.

(*R*)-1-(((S)-1-((3,5-bis(trifluoromethyl)benzyl)oxy)-3-(1H-indol-3-yl)-1-oxopropan-2-yl)amino)-1-oxopropan-2-aminium 2,2,2-trifluoroacetate, TFA*NH_2_-D-Ala-L-Trp-O-CH_2_-3,5(CF_3_)_2_Ph, 2b, white powder, yield 48%, purity > 95% (HPLC-UV), ESI-MS ion found m/z [M_base_+H]^+^: 502.10, [M_base_−H]^−^: 500.05, [M_sal_t−H]^−^: 614.00 calculated [M_base_+H]^+^: 502.16, [M_base_−H]^−^: 500.14, [M_sal_t−H]^−^: 614.14; t_R_ = 11.40 min; ^1^H NMR (600 MHz, DMSO-*d*6) δ (ppm), 10.84 (1H, s), 8.84 (1H, d, *J* = 7.8 Hz), 8.07 (1H, s), 7.99 (2H, s), 7.88 (3H, bs), 7.46 (1H, d, *J* = 7.8 Hz), 7.29 (1H, d, *J* = 8.4 Hz), 7.11 (1H, d, *J* = 2.1 Hz), 7.02 (1H, m), 6.93 (1H, m), 5.29 (1H, d, *J* = 13.2 Hz), 5.22 (1H, d, *J* = 13.2 Hz), 4.70 (1H, m), 3.77 (1H, m), 3.25 (1H, m), 3.08 (1H, m), 1.12 (3H, d, *J* =7.0 Hz).

(*R*)-1-(((R)-1-(((S)-1-((3,5-bis(trifluoromethyl)benzyl)oxy)-3-(1H-indol-3-yl)-1-oxopropan-2-yl)amino)-1-oxopropan-2-yl)amino)-1-oxopropan-2-aminium 2,2,2-trifluoroacetate, TFA*NH_2_-D-Ala-D-Ala-L-Trp-O-CH_2_-3,5(CF_3_)_2_Ph, 3b, white powder, yield 32%, purity > 98% (HPLC-UV), ESI-MS ion found m/z [M_base_+H]^+^: 573.15, [M_base_−H]^−^: 571.05, [M_sal_t−H]^−^: 685.00 calculated [M_base_+H]^+^: 573.20, [M_base_−H]^−^: 571.18, [M_sal_t−H]^−^: 685.18; t_R_ = 11.62 min; ^1^H NMR (500 MHz, DMSO-*d*6) δ (ppm), 10.85 (1H, s), 8.51 (1H, d, *J* = 7.5 Hz), 8.44 (1H, d, *J* = 7.5 Hz), 8.07 (1H, s), 7.99 (2H, s), 7.89 (3H, bs), 7.48 (1H, m), 7.32 (1H, m), 7.15 (1H, d, *J* = 2.2 Hz), 7.05 (1H, m), 6.96 (1H, m), 5.30 (1H, d, *J* = 13.3 Hz), 5.21 (1H, d, *J* = 13.3 Hz), 4.58 (1H, m), 4.39 (1H, m), 3.81 (1H, m), 3.24 (1H, m), 3.09 (1H, m), 1.26 (3H, d, *J* =7.0 Hz), 1.09 (3H, d, *J* =7.1 Hz). ^13^C NMR (125 MHz, DMSO-*d*6) δ (ppm), 172.21, 172.03, 169.45, 158.31 (weak, probably q with ^2^*J*_CF_ ~ 30 Hz, consistent with TFA salt), 139.78, 136.54, 130.74 (q, ^2^*J_CF_* = 32.5 Hz), 128.87 (likely an unresolved quartet with ^3^*J*_CF_ ~4Hz), 127.40, 124.30, 123.67 (q, ^2^*J_CF_* ~272 Hz), 122.20 (likely an unresolved quartet with ^3^*J*_CF_ ~4Hz), 121.43, 118.84, 118.30, 111.90, 109.63, 65.02, 53.62, 48.48, 48.43, 27.45, 18.93, 17.58. A signal coming from the CF_3_ group of the trifluoroacetate anion not observed (expected around 117.8 as a quartet with ^1^*J*_CF_ ~300 Hz).

(*S*)-6-((1-((3,5-bis(trifluoromethyl)benzyl)oxy)-3-(1H-indol-3-yl)-1-oxopropan-2-yl)amino)-6-oxohexan-1-aminium 2,2,2-trifluoroacetate, TFA*NH_2_-Ahx-L-Trp-O-CH_2_-3,5(CF_3_)_2_Ph, 4b, off-white powder, yield 65%, purity > 98% (HPLC-UV), ESI-MS ion found m/z [M_base_+H]^+^: 544.15, [M_base_−H]^−^: 542.10, [M_sal_t−H]^−^: 656.05 calculated [M_base_+H]^+^: 544.21, [M_base_−H]^−^: 542.19, [M_sal_t−H]^−^: 656.19; t_R_ = 11.67 min; ^1^H NMR (500 MHz, DMSO-*d*6) δ (ppm), 10.84 (1H, s), 8.35 (1H, d, *J* = 7.1 Hz), 8.05 (1H, s), 7.95 (2H, s), 7.59 (3H, bs), 7.48 (1H, m), 7.32 (1H, m), 7.15 (1H, d, *J* = 2.0 Hz), 7.06 (1H, m), 6.97 (1H, m), 5.28 (1H, d, *J* = 13.5 Hz), 5.17 (1H, d, *J* = 13.5 Hz), 4.54 (1H, m), 3.19 (1H, m), 3.07 (1H, m), 2.70 (2H, m), 2.09 (2H, t, *J* = 7.6 Hz), 1.50–1.38 (4H, m), 1.20 (2H, m). ^13^C-NMR spectra of the corresponding hydrochloride salt are given in SM.

(*S*)-6-((6-((1-((3,5-bis(trifluoromethyl)benzyl)oxy)-3-(1H-indol-3-yl)-1-oxopropan-2-yl)amino)-6-oxohexyl)amino)-6-oxohexan-1-aminium 2,2,2-trifluoroacetate, TFA*NH_2_-Ahx-Ahx-L-Trp-O-CH_2_-3,5(CF_3_)_2_Ph, 5b, off-white powder, yield 57%, purity > 97% (HPLC-UV), ESI-MS ion found m/z [M_base_+H]^+^: 657.20, [M_base_−H]^−^: 655.10, [M_sal_t−H]^−^: 769.10 calculated [M_base_+H]^+^: 657.29, [M_base_−H]^−^: 655.27, [M_sal_t−H]^−^: 769.26; t_R_ = 11.73 min. ^1^H NMR (500 MHz, DMSO-*d*6) δ (ppm), 10.84 (1H, s), 8.32 (1H, d, *J* = 7.2 Hz), 8.04 (1H, s), 7.96 (2H, s), 7.69 (1H, t, *J* = 5.5 Hz), 7.60 (3H, bs), 7.48 (1H, m), 7.32 (1H, m), 7.15 (1H, d, *J* = 2.0 Hz), 7.05 (1H, m), 6.96 (1H, m), 5.28 (1H, d, *J* = 13.7 Hz), 5.17 (1H, d, *J* = 13.7 Hz), 4.53 (1H, m), 3.19 (1H, m), 3.08 (1H, m), 2.95 (2H, m), 2.76 (2H, m), 2.10–2.00 (4H, m), 1.49 (4H, m), 1.42 (2H, m), 1.34–1.22 (4H, m), 1.15 (2H, s). ^13^C-NMR spectra of the corresponding hydrochloride salt are given in SM.

#### 3.1.6. Acetylation Reaction (**1c**, **2c**, **3c**, **4c**, **5c**) 

To a cooled solution (0–5 °C) of *N^α^*-deprotected derivatives (**1b** or **2b** or **3b** or **4b** or **5b**, 1 equiv) and TMG (2 equiv) in DMF/DCM (1:1), Ac_2_O (2 equiv) was added and the reaction mixture was allowed to stir for half an hour at 5 °C and for the next hour at room temperature. After concentration, the reaction mixture was acidified with 10% aqueous citric acid and extracted with AcOEt. The organic phase was dried with anh. MgSO_4_, the solvent was removed in vacuo and the resulting oil was dried.

**3,5-bis(trifluoromethyl)benzyl acetyl-L-tryptophanate**, Ac-NH-L-Trp-O-CH_2_-3,5(CF_3_)_2_Ph, **1c** (L732,138 [16]), white powder, yield 98%, purity > 98% (HPLC-UV), ESI-MS ion found m/z [M+H]^+^: 473.10 calculated [M+H]^+^: 473.13; t_R_ = 18.61 min; ^1^H NMR (500 MHz, DMSO-*d*6) δ (ppm) 10.84 (1H, s), 8.41 (1H, d, *J* = 6.9 Hz), 8.05 (1H, s), 7.96 (2H, s), 7.47 (1H, m), 7.32 (1H, m), 7.16 (1H, d, *J* = 2.2 Hz), 7.06 (1H, m), 6.97 (1H, m), 5.28 (1H, d, *J* = 13.5 Hz), 5.18 (1H, d, *J* = 13.5 Hz), 4.53 (1H, m), 3.18 (1H, m), 3.07 (1H, m), 1.82 (3H, s). ^13^C NMR (125 MHz, DMSO-*d*6) δ (ppm), 172.35, 169.99, 139.99, 136.55, 130.72 (q, ^2^*J_CF_* = 32.5 Hz), 128.59 (broad, likely an unresolved quartet with ^3^*J*_CF_ ~4Hz), 127.44, 124.13, 123.68 (q, ^1^*J_CF_* = 270.8 Hz), 121.43, 122.02 (broad, likely an unresolved quartet with ^3^*J*_CF_ ~4Hz), 118.86, 118.29, 111.93, 109.82, 64.69, 53.97, 27.31, 22.58.

**3,5-bis(trifluoromethyl)benzyl acetyl-D-alanyl-L-tryptophanate**, Ac-NH-D-Ala-L-Trp-O-CH_2_-3,5(CF_3_)_2_Ph, **2c**, white powder, yield 61%, purity > 97% (HPLC-UV), ESI-MS ion found m/z [M+H]^+^: 544.15 calculated [M+H]^+^: 544.17; t_R_ = 17.72 min; ^1^H NMR (600 MHz, DMSO-*d*6) δ (ppm), 10.84 (1H, s), 8.29 (1H, d, *J* = 7.5 Hz), 8.03 (1H, s), 7.96 (2H, s), 7.9 (1H, d, *J* = 7.5 Hz), 7.44 (1H, d, *J* = 7.8 Hz), 7.28 (1H, m), 7.11 (1H, d, *J* = 2.1 Hz), 7.02 (1H, m), 6.92 (1H, m), 5.25 (1H, d, *J* = 13.2 Hz), 5.16 (1H, d, *J* = 13.2 Hz), 4.49 (1H, m), 4.26 (1H, m), 3.18 (1H, m), 3.06 (1H, m), 1.76 (3H, s), 1.01 (3H, d, *J* =7.1 Hz).

**3,5-bis(trifluoromethyl)benzyl acetyl-D-alanyl-D-alanyl-L-tryptophanate**, Ac-NH-D-Ala-D-Ala-L-Trp-O-CH_2_-3,5(CF_3_)_2_Ph, **3c**, white powder, yield 62%, purity > 98% (HPLC-UV), ESI-MS ion found m/z [M+H]^+^: 615.20 calculated [M+H]^+^: 615.21; t_R_ = 17.07 min; ^1^H NMR (600 MHz, DMSO-*d*6) δ (ppm), 10.83 (1H, s), 8.25 (1H, d, *J* = 7.4 Hz), 8.03 (1H, s), 7.98 (1H, d, *J* = 7.3 Hz), 7.96 (2H, s), 7.86 (1H, d, *J* = 7.75 Hz), 7.43 (1H, m), 7.28 (1H, m), 7.13 (1H, d, *J* = 2.1 Hz), 7.02 (1H, m), 6.92 (1H, m), 5.23 (1H, d, *J* = 13.3 Hz), 5.16 (1H, d, *J* = 13.3 Hz), 4.50 (1H, m), 4.24 (1H, m), 4.19 (1H, m), 3.18 (1H, m), 3.06 (1H, m), 1.79 (3H, s), 1.09 (3H, d, *J* =7.2 Hz), 1.05 (3H, d, *J* =7.2 Hz).

**3,5-bis(trifluoromethyl)benzyl (6-acetamidohexanoyl)-L-tryptophanate**, Ac-NH-Ahx-L-Trp-O-CH_2_-3,5(CF_3_)_2_Ph, **4c**, white powder, yield 63%, purity > 98% (HPLC-UV), ESI-MS ion found m/z [M+H]^+^: 586.15 calculated [M+H]^+^: 586.22; t_R_ = 17.58 min; ^1^H NMR (600 MHz, DMSO-*d*6) δ (ppm), 10.82 (1H, s), 8.31 (1H, d, *J* = 7.0 Hz), 8.02 (1H, s), 7.93 (2H, s), 7.71 (1H, bs), 7.44 (1H, m), 7.29 (1H, m), 7.12 (1H, d, *J* = 2.0 Hz), 7.05 (1H, m), 6.96 (1H, m), 5.25 (1H, d, *J* = 13.6 Hz), 5.14 (1H, d, *J* = 13.6 Hz), 4.50 (1H, m), 3.16 (1H, m), 3.04 (1H, m), 2.91 (2H, m), 2.04 (2H, t, *J* = 7.3 Hz), 1.74 (3H, s), 1.38 (2H, m), 1.27 (2H, m), 1.11 (2H, m). ^13^C NMR (125 MHz, DMSO-*d*6) δ (ppm), 172.87, 172.41, 169.30, 139.98, 136.54, 130.71 (q, ^2^*J_CF_* ~ 32.5 Hz), 128.67 (likely an unresolved quartet with ^3^*J*_CF_ ~4 Hz), 127.45, 124.13, 123.68 (q, ^1^*J_CF_* ~ 272 Hz), 122.07 (likely an unresolved quartet with ^3^*J*_CF_ ~4 Hz), 121.41, 118.82, 118.32, 111.91, 109.91, 64.73, 53.83, 35.28, 29.36, 27.28, 26.45, 25.26, 23.04. C_ε_ signal of Ahx overlapped by the DMSO-d6 signal at around 40 ppm.

**3,5-bis(trifluoromethyl)benzyl (6-(6-acetamidohexanamido)hexanoyl)-L-tryptophanate**, Ac-NH-Ahx-Ahx-L-Trp-O-CH_2_-3,5(CF_3_)_2_Ph, **5c**, white powder, yield 68%, purity > 98% (HPLC-UV), ESI-MS ion found m/z [M+H]^+^: 699.25 calculated [M+H]^+^: 699.30; t_R_ = 17.01 min; ^1^H NMR (500 MHz, DMSO-*d*6) δ (ppm), 10.84 (1H, s), 8.32 (1H, d, *J* = 7.4 Hz), 8.04 (1H, s), 7.96 (2H, s), 7.75 (1H, bs), 7.66 (1H, t, *J* = 5.6 Hz), 7.47 (1H, m), 7.32 (1H, m), 7.15 (1H, d, *J* = 2.3 Hz), 7.05 (1H, m), 6.96 (1H, m), 5.28 (1H, d, *J* = 13.5 Hz), 5.17 (1H, d, *J* = 13.5 Hz), 4.53 (1H, m), 3.19 (1H, m), 3.07 (1H, m), 3.02–2.92 (4H, m), 2.07 (2H, t, *J* = 7.4 Hz), 2.01 (2H, t, *J* = 7.4 Hz), 1.77 (3H, s), 1.49–1.27 (8H, m), 1.23–1.10 (4H, m). ^13^C NMR (125 MHz, DMSO-*d*6) δ (ppm), 172.87, 172.41, 172.23, 169.31, 139.98, 136.54, 130.71 (q, ^1^*J_CF_* ~32.5 Hz), 128.65 (likely an unresolved quartet with ^3^*J*_CF_ ~4Hz), 127.45, 124.12, 123.97 (q, ^1^*J_CF_* ~272 Hz), 122.05 (likely an unresolved quartet with ^3^*J*_CF_ ~4Hz), 121.41, 118.82, 118.32, 111.91, 109.91, 64.73, 53.83, 38.85, 38.69, 35.81, 35.29, 29.39, 27.28, 26.58, 26.45, 25.51, 25.26, 23.04. C_ε_ signal of one of the Ahx residues overlapped by the DMSO-d6 signal at around 40 ppm.

### 3.2. Synthesis of Conjugates and Radioconjugates

#### 3.2.1. General

All applied chemicals and solvents were purchased as a reagent grade (Sigma-Aldrich/Merck, Darmstadt, Germany) and applied without further purification. DOTA-NHS ester (1,4,7,10-tetraazacyclododecane-1,4,7,10-tetraacetic acid mono-*N*-hydroxysuccinimide ester) was purchased from CheMatech, Dijon, France. [^68^Ga]GaCl_3_ was eluted from the commercially available ^68^Ge/^68^Ga generator (Eckert & Ziegler, Berlin, Germany), while the solution of [^177^Lu]LuCl_3_ in 0.04 M HCl was purchased from Radioisotope Centre POLATOM, National Centre for Nuclear Research, Otwock-Świerk, Poland. ^68^Ge/^68^Ga generator eluates were obtained by semi-automated syringe pump that enables convenient and safe fractioning, allowing the use of fractions with the highest radionuclide content in the labeling reaction; no other processing was applied.

The HPLC conditions and gradient were set as follows: a semi-preparative Phenomenex Jupiter Proteo column (C12, reversed phase) 4 μm, 90 Å, 250 × 10 mm, with UV/Vis (220 nm) or/and radio γ-detection at gradient elution: 0–20 min 20 to 80% solvent B; 20–30 min 80% solvent B; 2 mL/min.; solvent A: 0.1% (*v*/*v*) trifluoroacetic acid (TFA) in water; and solvent B: 0.1% (*v*/*v*) TFA in acetonitrile. Sep-Pack^®^ Classic Short C18 Cartridges were purchased from WATERS, Milford, MA, USA.

Mass spectra were measured on the Bruker 3000 Esquire mass spectrometer equipped with electrospray ionization (Bruker, Billerica, MA, USA).

#### 3.2.2. General Procedure of Syntheses of Conjugates with DOTA

A derivative with the primary amino group (**1b**–**5b**) (1 equiv.) and the DOTA-NHS ester (1 equiv.) were dissolved in DMF purged from oxygen with technical nitrogen and supplemented with a 3 equiv. of triethylamine. The reaction mixture was vigorously stirred overnight at about 50 °C. The progress of the reaction was monitored by HPLC. The crude reaction mixture was evaporated, dissolved in the HPLC mobile phase, purified by the HPLC method and lyophilized. The isolated main product was identified as a DOTA conjugate (**1d**–**5d**, >90% reaction yield) by MS analysis confirmation.

MS: Calculated monoisotopic mass for **1d**, C_36_H_42_F_6_N_6_O_9_: 816.74; found: 817.38 *m*/*z* [M + H]^+^
MS: Calculated monoisotopic mass for **2d**, C_39_H_47_F_6_N_7_O_10_: 887.82; found: 888.35 *m*/*z* [M + H]^+^
MS: Calculated monoisotopic mass for **3d**, C_42_H_52_F_8_N_8_O_11_: 958.90; found: 959.40 *m*/*z* [M + H]^+^
MS: Calculated monoisotopic mass for **4d**, C_42_H_53_F_6_N_7_O_10_: 929.90; found: 930.38 *m*/*z* [M + H]^+^
MS: Calculated monoisotopic mass for **5d**, C_48_H_64_F_6_N_8_O_11_: 1043.06; found: 1043.58 *m*/*z* [M + H]^+^

#### 3.2.3. ^68^Ga Radiolabelling

The ^68^Ga radiolabelling of DOTA conjugates for physiochemical evaluation was performed in accordance with the following procedure: 220 µL of [^68^Ga]GaCl_3_ in 0.1 M HCl from the ^68^Ge/^68^Ga generator (42.3–54.5 MBq) was added into the solution of 25 nmol of the selected conjugate in 300 µL of a 0.2 M acetate buffer (pH = 4.5) and heated for 10 min at 95 °C. Subsequently, radioconjugate was purified using Sep-Pack^®^ Classic Short C18 cartridge in accordance with the producer recommendations, thereby obtaining an easily vaporized ethanolic solution of radioconjugate. The effectiveness of the purification was monitored by HPLC.

#### 3.2.4. ^177^Lu Radiolabelling

The ^177^ Lu radiolabelling of DOTA conjugates for physiochemical and in vitro evaluation was performed in accordance with the following procedure: 2.5–5 MBq of a [^177^Lu]LuCl_3_ solution in 0.04 M HCl (1.5–5.7 µL) was added into the solution of 2.5 nmol of the selected conjugate in 200 µL of a 0.02 M acetate buffer (pH 4.5) and heated for 10 min at 95 °C. Subsequently, radioconjugate was purified using Sep-Pack^®^ C18 cartridge in accordance with the producer recommendations, thereby obtaining an easily vaporized ethanolic solution of radioconjugate. The effectiveness of the purification was monitored by HPLC.

#### 3.2.5. Preparation of Non-Radioactive References

The non-radioactive Ga labelling of DOTA conjugates was performed in accordance with the following procedure: 220 µL of a solution of 20 mM GaCl_3_ in 0.1 M HCl was added into the solution of 50 nmol of the selected conjugate in 300 µL of a 0.2 M acetate buffer (pH = 4.5) and heated for 10 min at 95 °C. Subsequently, reaction product was purified by the HPLC method, lyophilized and characterized by mass spectrometry.

MS: Calculated for monoisotopic mass **Ga-1d**, C_41_H_50_F_7_N_9_O_10_Ga: 883.20 and 885.20; found: 883.31 and 885.28 *m*/*z* [M]^+^
MS: Calculated for monoisotopic mass **Ga-2d**, C_41_H_49_F_7_N_10_O_11_Ga: 954.24 and 956.24; found: 954.33 and 956.65 *m*/*z* [M]^+^
MS: Calculated for monoisotopic mass **Ga-3d**, C_43_H_53_F_7_N_10_O_11_Ga: 1025.28 and 1027.27; found: 1025.37 and 1027.38 *m*/*z* [M^+^]
MS: Calculated for monoisotopic mass **Ga-4d**, C_42_H_52_F_7_N_9_O_10_Ga: 996.29 and 998.28; found: 996.39 and 998.38 *m*/*z* [M]^+^
MS: Calculated for monoisotopic mass **Ga-5d**, C_43_H_54_F_7_N_9_O_10_Ga: 1109.37 and 1111.37; found: 1109.09 and 1111.20 *m*/*z* [M]^+^

### 3.3. Plasma Stability Study

Human serum aliquots were isolated and purified at the Centre of Radiobiology and Biological Dosimetry, INCT Warsaw, Poland. A solution of isolated radioconjugate in 100 µL of 0.1M PBS buffer (pH 7.40) was added into 900 µL of human serum and incubated at 37 °C for 4.5 h (in case of ^68^Ga-radioconjugates) or 2 days (^177^Lu-radioconjugates). At specific time points, 400 µL of the incubated mixture was added into 500 µL of ethanol, vigorously stirred to precipitate serum proteins and centrifuged (13,500 rpm for 5 min) to separate the supernatant for HPLC analysis.

### 3.4. Lipophilicity Determination

A solution of isolated radioconjugate (approximately 0.5 MBq for ^177^Lu-radioconjugates or 5 MBq for ^68^Ga-radioconjugates) in 500 µL of 0.1 M PBS buffer (pH 7.40) and 500 µL of *n*-octanol was vigorously stirred and centrifuged (13,500 rpm for 5 min) to separate the immiscible phases. Then, the radioactivity of samples from both phases were measured using a well-type NaI(Tl) detector. The distribution coefficient, D, was calculated dividing the radioactivity of the radioconjugate in the organic phase to that in the aqueous phase. Each experiment was performed in triplicate and averaged. In parallel, the aqueous phases were analysed by HPLC to confirm the stability of studied radioconjugate during the experiment period. The lipophilicity value of each radioconjugates was expressed as the logarithm of its D value.

### 3.5. Cell Culture

The CHO-K1 cells with a stable overexpression of the human NK1 receptor, denoted further on as hNK1-CHO cells, were obtained as a gift from Dr. Attila Keresztes and Dr. John M. Streicher [29]. The cells were maintained in a humidified atmosphere with 5% CO_2_ at 37 °C, in Ham’s F12 medium (Corning, Corning, NY, USA) supplemented with 10% Foetal Bovine Serum (CytoGen, Wetzlar, Germany) and 400 μg/mL G418 (Corning, Corning, NY, USA).

### 3.6. hNK1-CHO Membrane Preparation

When the cells reached approximately 90% confluence, they were harvested by treatment with 0.05% trypsin/EDTA (Corning, Corning, NY, USA). After centrifugation at 1500 rpm for 5 min, the cells were homogenized in a glass tissue homogenizer in ice-cold 50 mM Tris-HCl buffer (pH 7.4) with 5 mM MnCl_2_. The preparation was collected by centrifugation at 13,000 rpm for 25 min at 4 °C, and then the pellet was suspended in 50 mM Tris-HCl buffer. The homogenates were stored at −80 °C for later use. The BCA assay (Thermo Scientific, Waltham, MA, USA) was used to determine the protein concentration in the homogenates.

### 3.7. Competitive Binding Assays

The binding affinity of the tested compounds for human and rat NK1R was determined in competitive radioligand binding assays following the method previously described [15]. As a source of the receptor proteins, homogenates obtained from rat brains (obtained as described in Ref [30]) or homogenates made from hNK1-CHO cells were used. Different concentrations of the test compounds were incubated with the appropriate membrane preparation (in concentrations of 1.125 mg/mL or 3.8 mg/mL for human and rat preparations, respectively) and 1 nM [2-Prolyl-3,4-^3^H]-(Sar^9^,Met(O_2_)^11^)-Substance P (PerkinElmer, Waltham, MA, USA) in the assay buffer (50 mM Tris–HCl (pH 7.4), 5 mM MnCl_2_, bovine serum albumin (BSA) (0.1 mg/mL), bacitracin (100 μg/mL), bestatin (30 μM), phenylmethylsulphonyl fluoride (30 μg/mL) and captopril (10 μM)). The final reaction volumes were 250 μL and 1 mL, for the human and rat NK1R determinations, respectively. Substance P (10 μM, Tocris, United Kingdom) was used to measure the non-specific binding. The binding reaction mixtures were incubated in a shaking water bath at 25 °C for 60 min. The reaction was terminated by rapid filtration through Whatman GF/B filter (Gaithersburg, MD) presoaked in 0.5% polyethyleneimine and washed three times with 2 mL of cold saline. The filters were placed in 24-well plates and immersed in a Betaplate Scint scintillation solution (Perkin Elmer, Waltham, MA, USA). The filter-bound radioactivity was determined by scintillation counter MicroBeta LS, Trilux (PerkinElmer, Waltham, MA, USA).

The displacement curves were drawn and the mean IC_50_ values were determined with standard deviations (GraphPad Prism version 8.0, San Diego, CA, USA). At least three independent experiments (carried out in duplicate) were performed.

### 3.8. Saturation Binding Assays

The saturation binding studies of ^177^Lu-radioconjugates were performed using transfected CHO-NK1R cell line, and all radioconjugates were obtained according to the common labelling protocol given above with the same specific activity. In brief, 10^5^ cells per well were seeded into 24-well plates and incubated 24 h before the experiment. Just before the assay, cells were washed with 37 °C Dulbecco’s PBS and then incubated with different concentrations of radioconjugate (0.2–25 nM at a final volume) with or without 1000-fold molar excess of blocker (NK1R high affinity nonpeptide antagonist aprepitant) for 60 min at 37 °C. After that time, the assay medium was collected into plastic tubes and cells were washed twice with cold Dulbecco’s PBS. Subsequently, cells were lysed using 1M NaOH and collected into plastic tubes for radioactivity measurement.

The radioactivity of collected medium and lysed cells was measured using a Wizard^2^ 2-Detector Gamma Counter (PerkinElmer, Waltham, MA, USA) with the energy window of 50–500 keV. The study data came from three independent experiments carried out in duplicate, while the results, presented as the Kd and BMAX with SD, were calculated using a total and nonspecific binding (one site) nonlinear regression curve fit (GraphPad Prism version 8.0, GraphPad, San Diego, CA, USA).

### 3.9. Molecular Docking

Molecular docking was executed using AutoDock 4.2.6. [31]. The structure of **1c** (separate for *cis* and *trans* conformers around the amide bond) was optimized at the B3LYP/6-31G(d,p) level by using Gaussian09 [32]. Both *cis* and *trans* conformers of 1c were used for docking. The structures of further analogues were built by manually expanding the structure of **1c** in the binding site of hNK1R, whereafter the optimization was performed by local search routine in AutoDock 4.2.6 [31].

The ligands and the protein structure were processed in AutoDock Tools 4 [31] with standard routines. Full ligand flexibility (except for amide bonds) was allowed. The receptor was treated as rigid. In order to take into account side-chain flexibility in the binding site, in the first stages of docking three separate hNK1R structures (6HLL, 6HLO and 6HLP [25]) were used. With the initial results in hand, it was decided to use only the 6HLO structure.

The receptor structures were the refined models provided by the GPCRdb service [33]. This was chosen in order to have the mutated residues replaced with native ones, as well as to have side chains missing in the original PDB structures supplemented. Before docking, the model coordinates were transformed to match the coordinates of corresponding models in OPM database [34]. This was carried out to facilitate embedding in lipid membrane for the purposes of molecular dynamics.

The docking boxes encompassed the orthosteric binding site of hNK1R and the extracellular outlet of the binding sites. The grids were calculated with AutoGrid 4 [31].

For global docking, default AutoDock parameters were used. For local searches, we used the following settings: 300 individuals in population, 500 iterations of the Solis-Wets local search, the *sw*_*rho* parameter of the local search space set to 100.0 and 1000 local search runs. The calculations were repeated several times. The local docking results were clustered, and structures from the best scored clusters were taken for further analyses.

### 3.10. Molecular Dynamics

The receptor–ligand complexes as obtained by docking were embedded in POPC membrane (128 lipid molecules) and solvated with TIP3P water (about 13000 water molecules, TIP3P). Na^+^ and Cl^−^ ions (0.154 M concentration) were added too. The systems were prepared by using the CHARMM-GUI service [35]. CHARMM 36 force field was used for the proteins, lipids, water and ions, while the ligands were modelled using CHARMM CGenFF [36].

Molecular dynamics simulations were run in GROMACS 5.1.2 [37]. The complexes were minimized and equilibrated. The production runs were obtained using the following parameters: NPT ensemble, temperature = 303.15 K, integration step = 2 fs, cut-off scheme Verlet, Nose–Hoover thermostat, Parrinello–Rahman barostat, LINCS H-bonds constraints. For each system, 3 runs of 150 ns production length were obtained.

For analysing conformational behaviour of the complexes, the trajectories for each complex were concatenated and superposed on a common reference snapshot. The superposition was based on backbone atoms of the helical part of the receptors. The root-mean-square deviations of the atomic positions of the protein (in helical part), the ligand or ligand’s parts were monitored over simulation times (using built-in GROMACS tools).

The molecular graphics were prepared in open-source PyMol [38] and in Biovia Discovery Studio Visualizer v. 19 [39].

## 4. Conclusions

The work reported herein shows that a small molecular NK1R antagonist, L732,138, may be a good basis for creating radioconjugates. Such radioconjugates (containing therapeutic radionuclides) could be useful agents for the targeted radionuclide therapy of glioblastoma multiforme, for the locoregional administration. If labelled with diagnostic radionuclides, they might also be applied for diagnostic purposes.

In the first stage of our work, we showed that novel (expanded) L732,138 analogues are good hNK1R binders, practically regardless of the length of the linker used. None of the evaluated linkers exhibited unequivocally negative or unequivocally positive effects on the receptor affinity. The best analogues (e.g., **5a**) showed some improvement compared to the parent analogue.

The obtained radioconjugates, containing DOTA chelator coupled to L732,138 and its four analogues with different linker lengths, after labelling with ^68^Ga and ^177^Lu, turned out to be fairly lipophilic (logD_7.4_). Unfortunately, they showed rather limited stability in human plasma, with degradation occurring within a few hours. However, it is not necessarily disqualifying, as their prospective application is for the locoregional administration into the post-operation cavity where the enzymatic activity might be expected to be negligible.

Evaluation of the receptor affinity for the DOTA-conjugates and their complexes with Ga^3+^ and Lu^3+^ showed that neither the chelator nor the metal negatively impact the hNK1R binding. For the ^177^Lu-radioconjugates, we found K_d_ values in the range 2–10 nM, which is comparable to the K_d_ found for the reference SP-based radioconjugate. Very importantly, in the saturation assays for the novel radioconjugates, one observes a higher binding capacity (B_MAX_ values) than in the case of the reference radiopharmaceutical. This is probably due to the use of a small molecular, lipophilic structure as the vector.

By the means of molecular modelling, a binding mode (consistent with the previous experimental hints) for the parent L732,138 was established. Interestingly, this compound’s binding is predicted to be stabilized by exclusively apolar contacts. On the contrary, in the cases of other analogues, polar interactions are of importance. Modelling also sheds light on the binding mode of the conjugate **1d** (no linker), whose accommodation in the binding site is predicted to be associated with the flexibility of the receptor. The results of the modelling and of the experimental part are going to guide future SAR work.

The work reported herein is a further step towards the development of NK1R-targeted radioconjugates based on small molecular vectors. Of course, these results do not allow for the direct application of our radioconjugates in medicine. Further experimental work on these analogues or some novel ones is required (including in vivo experiments).

It is to be noted that NK1R-targeting conjugates and polyfunctional ligands are also contemplated outside of the radiopharmacy, e.g., in the research on multitarget analgesics [40,41,42] or in the efforts towards therapeutic gene delivery [43,44]. In these areas, the novel NK1R ligands that were reported herein and the SAR data may be of use too.

## Figures and Tables

**Table 1:** NK1R binding affinity of the synthesized analogues.

Compound	Linker Length	Linker	*N*-Terminus	IC_50_ (Mean ± SD) ^1^	
				rNK1R	hNK1R
	(n Atoms)			(nM)	(nM)
**1a**	0	none	Boc	2928 ± 1518	180.4 ± 112.4
**1b**	0	none	H	>10000	358.9 ± 132.0
**1c** L732,138 ^2^ (parent)	0	none	Ac	2879 ± 999	25.6 ± 15.9
**2a**	3	-DAla-	Boc	1532 ± 279	135.0 ± 108.3
**2b**	3	-DAla-	H	4681 ± 1322	25.4 ± 12.5
**2c**	3	-DAla-	Ac	2280 ± 703	25.3 ± 0.4
**3a**	6	-DAla-DAla-	Boc	745 ± 311	13.9 ± 11.2
**3b**	6	-DAla-DAla-	H	4007 ± 1373	80.8 ± 59.7
**3c**	6	-DAla-DAla-	Ac	1141 ± 289	13.5 ± 12.1
**4a**	7	-Ahx-	Boc	1658 ± 1051	60.7 ± 31.9
**4b**	7	-Ahx-	H	1399 ± 319	35.4 ± 23.6
**4c**	7	-Ahx-	Ac	1951 ± 641	83.1 ± 69.3
**5a**	14	-Ahx-Ahx-	Boc	762 ± 295	4.5 ± 2.8
**5b**	14	-Ahx-Ahx-	H	572 ± 41	20.4 ± 10.2
**5c**	14	-Ahx-Ahx-	Ac	655 ± 120	14.3 ± 5.2
**Reference compounds**					
**Substance P**	-	-	-	3.6 (*n* = 1)	30.7 ± 4.2 (*n* = 2)
**Aprepitant**	-	-	-	130 ± 60 [15]	27.7 (*n* = 1)

^1^ IC_50_, half maximal inhibitory concentration, mean of three independent experiments carried out in duplicate with the standard deviation, unless stated otherwise. ^2^ hNK1R binding affinity in the original report, IC_50_ = 1.6 nM [16].

**Table 2:** Retention times (t_R_) of stable gallium conjugates and ^68^Ga-radioconjugates.

Stable Ga-Conjugate	t_R_ ^1^	^68^Ga-Radioconjugate	t_R_
**Ga-1d**	17.9 min	**[^68^Ga]Ga-1d**	18.2 min
**Ga-2d**	17.7 min	**[^68^Ga]Ga-2d**	18.0 min
**Ga-3d**	17.6 min	**[^68^Ga]Ga-3d**	17.8 min
**Ga-4d**	18.1 min	**[^68^Ga]Ga-4d**	18.3 min
**Ga-5d**	18.2 min	**[^68^Ga]Ga-5d**	18.3 min

^1^ Gradient elution: 0–20 min 20 to 80% solvent B; 20–30 min 80% solvent B; 2 mL/min.; solvent A: 0.1% (*v*/*v*) TFA in H_2_O; and solvent B: 0.1% (*v*/*v*) TFA in ACN.

**Table 3:** LogD_7.4_ values of all radioconjugates determined in *n*-octanol/PBS buffer system.

Radioconjugate	logD_7.4_ (Mean ± SD) ^1^	
	^68^Ga-	^177^Lu-
**1d-**	−0.085 ± 0.015	0.400 ± 0.004
**2d-**	−0.06 ± 0.02	0.704 ± 0.005
**3d-**	−0.259 ± 0.017	0.619 ± 0.007
**4d-**	0.071 ± 0.023	0.866 ± 0.019
**5d-**	−0.466 ± 0.016	0.294 ± 0.013

^1^ logD values are presented as means of three independent experiments carried out in duplicate with the standard deviation.

**Table 4:** NK1R binding affinity of the synthesized conjugates (competitive binding assays).

Compound	Linker Length	Linker	*N*-Terminus	IC_50_ (Mean ± SD) ^1^	
				rNK1R	hNK1R
				**(nM)**	**(nM)**
**1d**	0	none	DOTA	n/d ^2^	7.2 ± 1.4
**Ga-1d**	0	none	Ga-DOTA	112 ± 3	5.1 ± 2.0
**2d**	3	-DAla-	DOTA	1536 ± 231	21.4 ± 5.7
**Ga-2d**	3	-DAla-	Ga-DOTA	747 ± 348	38.7 ± 22.9
**3d**	6	-DAla-DAla-	DOTA	1270 ± 415	27.1 ± 1.1
**Ga-3d**	6	-DAla-DAla-	Ga-DOTA	2393 ± 1114	14.6 ± 2.3
**4d**	7	-Ahx-	DOTA	n/d ^2^	8.7 ± 3.2
**Ga-4d**	7	-Ahx-	Ga-DOTA	1000 ± 71	15.5 ± 2.0
**5d**	14	-Ahx-Ahx-	DOTA	830 ± 236	4.2 ± 0.8
**Ga-5d**	14	-Ahx-Ahx-	Ga-DOTA	411 ± 183	8.7 ± 2.4

^1^ IC_50_, half maximal inhibitory concentration, mean of three independent experiments carried out in duplicate with the standard deviation, ^2^ n/d—not determined.

**Table 5:** The results of saturation binding assays towards the hNK1Rr for the novel ^177^Lu-radioconjugates, compared to the reference derivative of Substance P.

Radioconjugate	K_d_ ± SD ^1^ (nM)	Ratio to SP	B_MAX_ ± SD ^1^ (nM)	Ratio to SP
**[^177^Lu]Lu-DOTA-[Thi^8^,Met(O_2_)^11^]SP (reference)**	6.15 ± 0.64	1.00	0.929 ± 0.027	1.00
**[^177^Lu]Lu-1d**	6.98 ± 0.69	1.13	0.762 ± 0.046	0.82
**[^177^Lu]Lu-2d**	10.52 ± 0.77	1.71	2.497 ± 0.046	2.69
**[^177^Lu]Lu-3d**	2.002 ± 0.051	0.33	0.5939 ± 0.0063	0.64
**[^177^Lu]Lu-4d**	5.40 ± 0.30	0.88	2.363 ± 0.060	2.54
**[^177^Lu]Lu-5d**	8.70 ± 0.46	1.42	4.93 ± 0.26	5.31

^1^ Values are presented as means of three independent experiments carried out in duplicate with the standard deviation.

## Data Availability

The data presented in this study are available on request from the corresponding author.

## Supplementary Materials

The following are available online at https://www.mdpi.com/article/10.3390/ijms23031214/s1.
