# Supplementary material for: Novel NK1R-Targeted 68Ga-/177Lu-Radioconjugates with Potential Application against Glioblastoma Multiforme: Preliminary Exploration of Structure–Activity Relationships
Source: Int J Mol Sci. 2022 Jan 21;23(3):1214. doi: 10.3390/ijms23031214 (PMC8835841; doi:10.3390/ijms23031214)
Supplement: Supplementary file 1 [file ijms-23-01214-s001.zip › ijms-1509924-supplementary.pdf]

Supplementary Materials

to

Novel NK1R-targeted ^{68}Ga-/^{177}Lu-radioconjugates with potential application against glioblastoma multiforme: preliminary exploration of Structure-Activity Relationship

by

Joanna Matalińska,[1,†] Katarzyna Kosińska,[1,†] Paweł K. Halik,[2] Przemysław Koźmiński,[2] Piotr F. J. Lipiński,[1,*] Ewa Gniazdowska,[2] Aleksandra Misicka[1,3,*]

1 Department of Neuropeptides, Mossakowski Medical Research Institute Polish Academy of Sciences, 5 Pawińskiego Street, PL 02-106, Warsaw, Poland; jmatalinska@imdik.pan.pl (J.M.), kkosinska@imdik.pan.pl (K.K.), plipinski@imdik.pan.pl (P.F.J.L.), misicka@chem.uw.edu.pl (A.M.).

2 Centre of Radiochemistry and Nuclear Chemistry, Institute of Nuclear Chemistry and Technology, 03-195 Warsaw, Poland; p.halik@ichtj.waw.pl (P.K.H.); p.kozminski@ichtj.waw.pl (P.K.); e.gniazdowska@ichtj.waw.pl (E.G.).

3 University of Warsaw, Faculty of Chemistry, 1 Pasteura Street PL 02-093, Warsaw, Poland; misicka@chem.uw.edu.pl (A.M.).

* Correspondence: plipinski@imdik.pan.pl (P.F.J.L.), misicka@chem.uw.edu.pl (A.M.).

† these authors contributed equally

Table of contents

SM-SYN: synthesis

^{13}C-NMR spectrum of **4b:HCl**.
^{13}C-NMR spectrum of **5b:HCl**

SM-STAB: stability

Figure SM-STAB-1. γ-radiochromatograms of **[^{68}Ga]Ga-2d** incubated with human serum at 1h, 2h and 4.5 h time points.
Figure SM-STAB-2. γ-radiochromatograms of **[^{177}Lu]Lu-2d** incubated with human serum at 1d and 2d time points.

SM-MOD: modelling

Figure SM-MOD-1. RMSD plot of the ligand position in the simulations of 1c/hNK1R complexes.
Figure SM-MOD-2. RMSD plot of the 3,5-bistrifluoromethylbenzyl fragment's position in the simulations of 1c/hNK1R complexes.
Figure SM-MOD-3. RMSD plot of the indole ring's position in the simulations of 1c/hNK1R complexes.
Figure SM-MOD-4. Superposition of **1c** (L732,138) in its supposed bioactive conformation (as found in MD simulations with the receptor) and of (5*S*)-3-[[3,5-bis(trifluoromethyl)phenyl]methyl]-5-(1H-indol-3-ylmethyl)oxazolidine-2,4-dione.
Figure SM-MOD-5. Superposition of the binding pose found for **1d** (pink) by docking to a rigid receptor and that for **1c** (white).
Figure SM-MOD-6. RMSD plot of the ligand position in the simulations of 1d/hNK1R complexes.
Figure SM-MOD-7. RMSD plot of the 3,5-bistrifluoromethylbenzyl fragment's position in the simulations of 1d/hNK1R complexes.
Figure SM-MOD-8. RMSD plot of the indole ring's position in the simulations of 1d/hNK1R complexes.
Figure SM-MOD-9. RMSD plot of the DOTA fragment's position in the simulations of 1d/hNK1R complexes.
Figure SM-MOD-10. Scheme of the interactions of **1d** with the NK1R as found in the MD simulations (simulation **1d-1**, T = 150.0 ns).
Figure SM-MOD-11. Scheme of the interactions of **1d** with the NK1R as found in the MD simulations (simulation **1d-2**, T = 150.0 ns).
Figure SM-MOD-12. Scheme of the interactions of **1d** with the NK1R as found in the MD simulations (simulation **1d-3**, T = 150.0 ns).
Figure SM-MOD-13. RMSD plot of the ligand position in the simulations of 5a/hNK1R complexes.
Figure SM-MOD-14. RMSD plot of the 3,5-bistrifluoromethylbenzyl fragment's position in the simulations of 5a/hNK1R complexes.
Figure SM-MOD-15. RMSD plot of the indole ring's position in the simulations of 5a/hNK1R complexes.
Figure SM-MOD-16. RMSD plot of the Boc-Ahx-Ahx fragment's position in the simulations of 5a/hNK1R complexes.
Figure SM-MOD-17. RMSD plot of the -Ahx1- (closer to Trp) fragment's position in the simulations of 5a/hNK1R complexes.
Figure SM-MOD-18. RMSD plot of the -Ahx2- (N-terminal) fragment's position in the simulations of 5a/hNK1R complexes.
Figure SM-MOD-19. Scheme of the interactions of **5a** with the NK1R as found in the MD simulations (simulation **5a-1**, T = 210.0 ns).
Figure SM-MOD-20. Scheme of the interactions of **5a** with the NK1R as found in the MD simulations (simulation **5a-2**, T = 210.0 ns).
Figure SM-MOD-21. Scheme of the interactions of **5a** with the NK1R as found in the MD simulations (simulation **5a-3**, T = 210.0 ns).
Figure SM-MOD-22. RMSD plot of the ligand position in the simulations of 5b/hNK1R complexes.
Figure SM-MOD-23. RMSD plot of the 3,5-bistrifluoromethylbenzyl fragment's position in the simulations of 5b/hNK1R complexes.
Figure SM-MOD-24. RMSD plot of the indole ring's position in the simulations of 5b/hNK1R complexes.
Figure SM-MOD-25. RMSD plot of the NH_2-Ahx-Ahx- fragment's position in the simulations of 5b/hNK1R complexes.
Figure SM-MOD-26. RMSD plot of the -Ahx1- (closer to Trp) fragment's position in the simulations of 5b/hNK1R complexes.
Figure SM-MOD-27. RMSD plot of the -Ahx2- (N-terminal) fragment's position in the simulations of 5b/hNK1R complexes.
Figure SM-MOD-28. Scheme of the interactions of **5b** with the NK1R as found in the MD simulations (simulation **5b-1**, T = 250.0 ns).
Figure SM-MOD-29. Scheme of the interactions of **5b** with the NK1R as found in the MD simulations (simulation **5b-2**, T = 250.0 ns).
Figure SM-MOD-30. Scheme of the interactions of **5b** with the NK1R as found in the MD simulations (simulation **5b-3**, T = 250.0 ns).

Figure SM-MOD-31. Compound **5b** in the hNK1R binding pocket. A snapshot from 5b-1 MD simulation (t=247.1 ns).

Figure SM-MOD-32. Compound **5b** in the hNK1R binding pocket. A snapshot from 5b-2 MD simulation (t=251.6 ns).

Figure SM-MOD-33. Compound **5b** in the hNK1R binding pocket. A snapshot from 5b-3 MD simulation (t=236.0 ns).

SM-SYN: Synthesis

TFA anions of **4b** and **5b** were exchanged for chloride anions to give hydrochlorides **4b:HCl** and **5b:HCl**. This was done by dissolving samples in 0.1 M_{aq} hydrochloric acid and lyophilization. The procedure was repeated thrice.

4b:HCl

^{13}C NMR (150 MHz, DMSO-*d6*) δ (ppm), 172.76, 172.42, 140.01, 136.53, 130.7 (q, $^{2}J_{CF}$ ~ 32.5 Hz), 128.64 (likely an unresolved quartet with $^{3}J_{CF}$ ~ 4Hz), 127.42, 124.18, 123.68 (q, $^{1}J_{CF}$ ~ 272 Hz), 121.39, 122.06 (likely an unresolved quartet with $^{3}J_{CF}$ ~ 4Hz), 118.81, 118.29, 111.92, 109.85, 64.73, 53.85, 38.99, 35.06, 27.27, 27.14, 25.82, 24.97.

5b:HCl

^{13}C NMR (150 MHz, DMSO-*d6*) δ (ppm), 172.91, 172.40, 172.15, 139.97, 136.53, 130.71 (q, $^{1}J_{CF}$ ~ 32.5 Hz), 128.63 (likely an unresolved quartet with $^{3}J_{CF}$ ~ 4Hz), 127.42, 124.15, 123.69 (q, $^{1}J_{CF}$ ~ 272 Hz), 122.04 (likely an unresolved quartet with $^{3}J_{CF}$ ~ 4Hz), 121.38, 118.80, 118.28, 111.92, 109.87, 64.72, 53.85, 39.04, 38.72, 35.54, 35.27, 27.16, 29.36, 27.25, 26.43, 25.95, 25.24. $C_{ε}$ signal of one of the Ahx residues overlapped by the DMSO-d6 signal at around 40 ppm.

SM-STAB: Stability

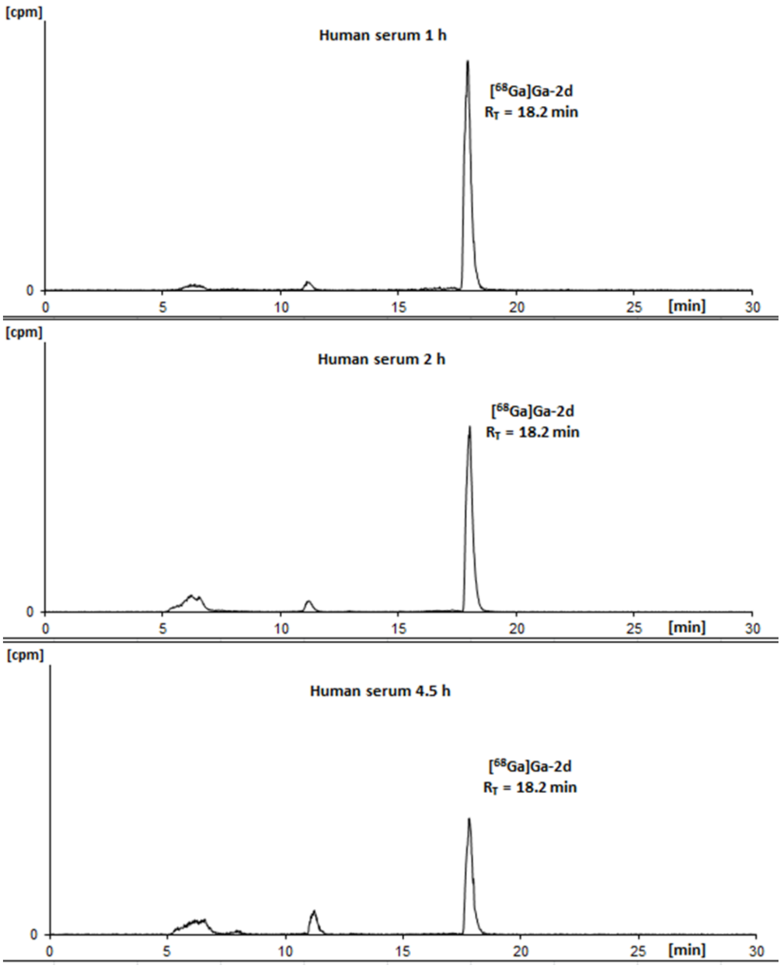

Figure SM-STAB-1. γ-radiochromatograms of **[^{68}Ga]Ga-2d** incubated with human serum at 1h, 2h and 4.5 h time points.

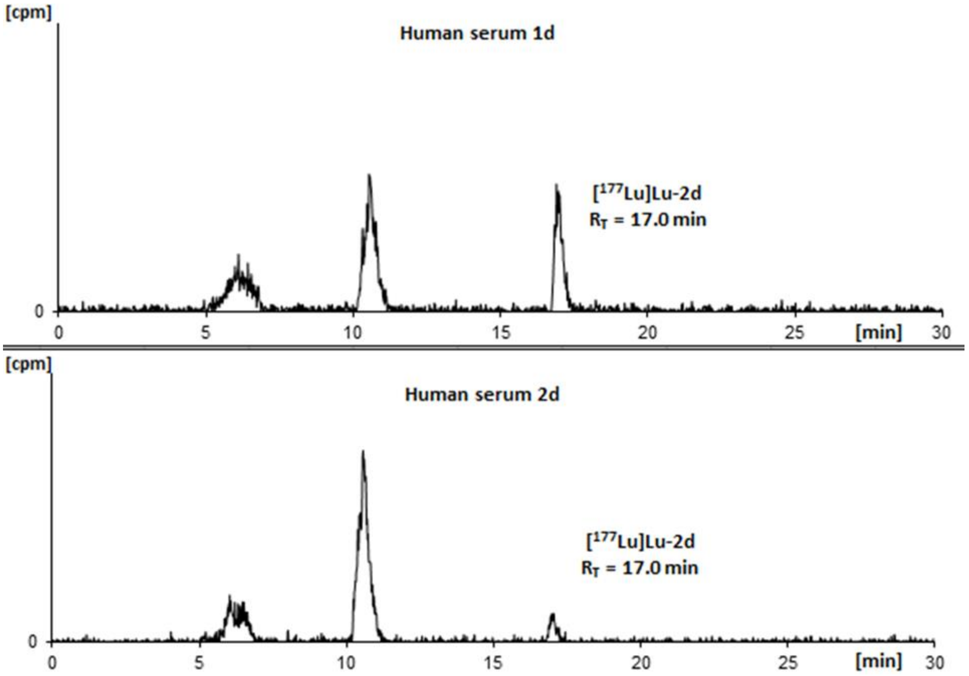

Figure SM-STAB-2. γ-radiochromatograms of **[^{177}Lu]Lu-2d** incubated with human serum at 1d and 2d time points.

.

SM-MOD: Molecular Modelling

Compound 1c

Ligand's position

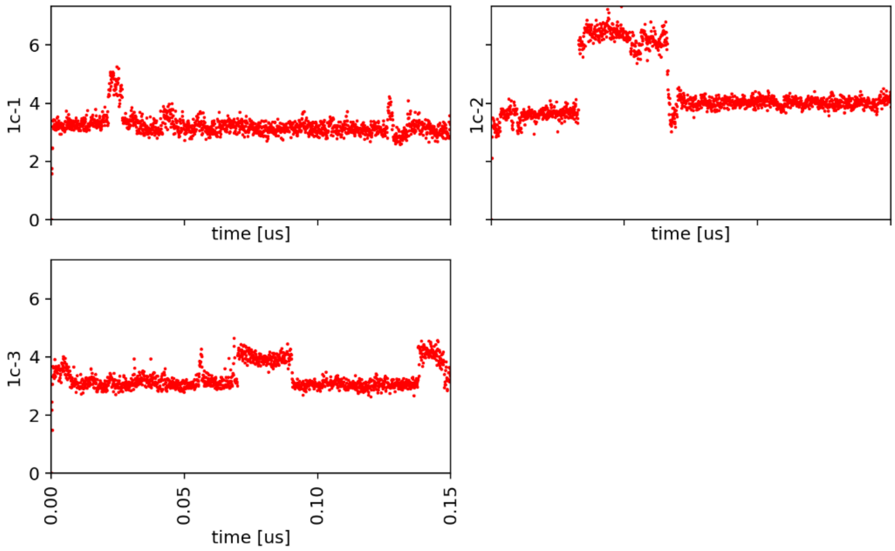

Figure SM-MOD-1. RMSD plot of the ligand position in the simulations of 1c/hNK1R complexes.

RMSD is given in Angstrems.

3,5-bistrifluoromethylbenzyl fragment's position

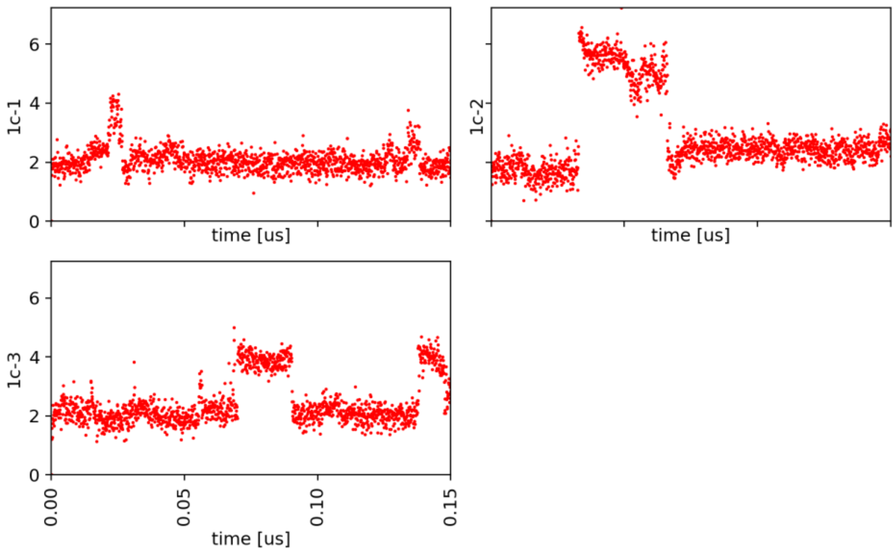

Figure SM-MOD-2. RMSD plot of the 3,5-bistrifluoromethylbenzyl fragment's position in the simulations of 1c/hNK1R complexes.

RMSD is given in Angstrems.

Indole ring's position

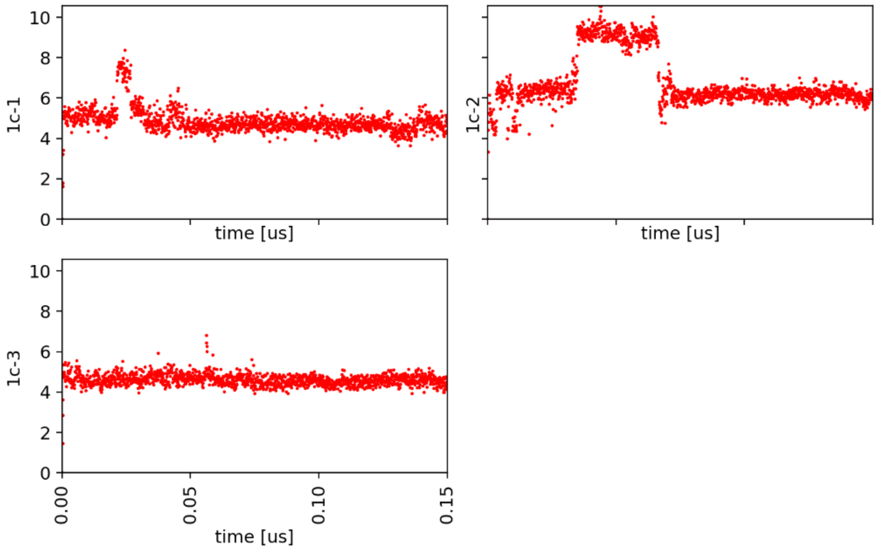

Figure SM-MOD-3. RMSD plot of the indole ring's position in the simulations of 1c/hNK1R complexes.

RMSD is given in Angstrems.

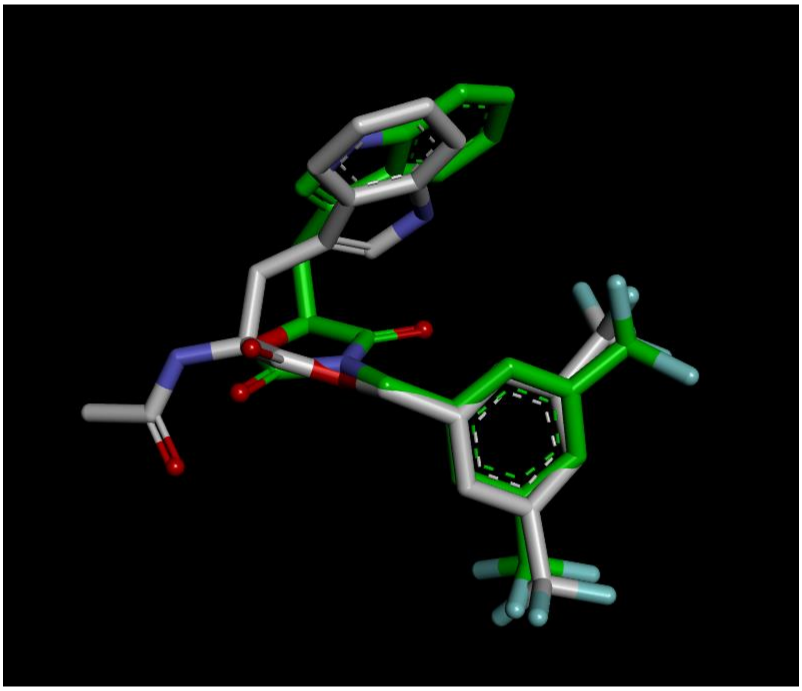

Figure SM-MOD-4. Superposition of **1c** (L732,138) in its supposed bioactive conformation (as found in MD simulations with the receptor) and of (5*S*)-3-[[3,5-bis(trifluoromethyl)phenyl]methyl]-5-(1H-indol-3-ylmethyl)oxazolidine-2,4-dione.

The rigidified derivative was described in: A. M. MacLeod, M. A. Cascieri, K. J. Merchant, S. Sadowski, S. Hardwicke, R. T. Lewis, D. E. MacIntyre, J. M. Metzger and T. M. Fong, J. Med. Chem., 1995, ***38****, 934–941.*

Compound 1d

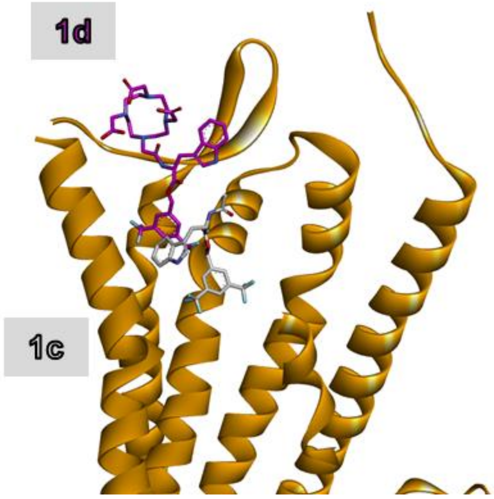

Figure SM-MOD-5. Superposition of the binding pose found for **1d** (pink) by docking to a rigid receptor and that for **1c** (white).

We would suspect such displaced positioning of the 3,5-bistrifluoromethylphenyl core to be associated with diminished binding affinity which is contrary to the experimental results.

Ligand's position

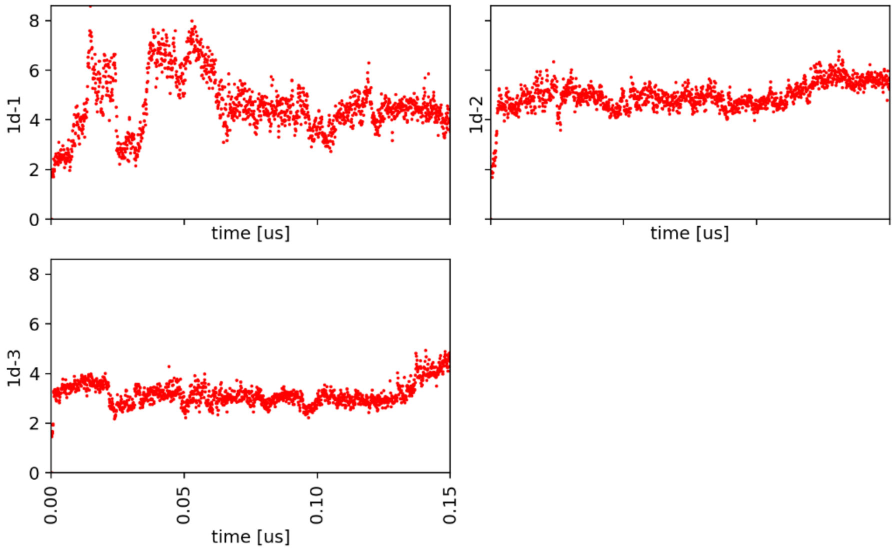

Figure SM-MOD-6. RMSD plot of the ligand position in the simulations of 1d/hNK1R complexes.

RMSD is given in Angstrems.

3,5-bistrifluoromethylbenzyl fragment’s position

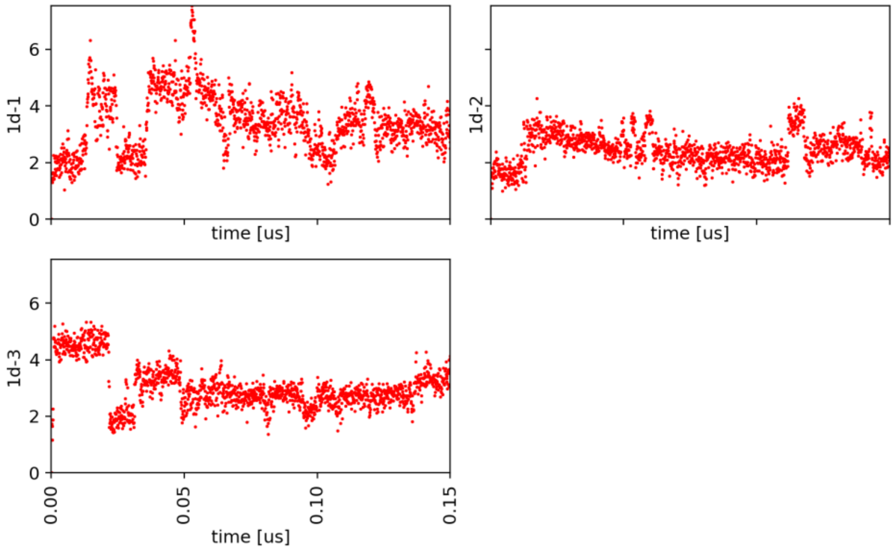

Figure SM-MOD-7. RMSD plot of the 3,5-bistrifluoromethylbenzyl fragment’s position in the simulations of 1d/hNK1R complexes.

RMSD is given in Angstrems.

Indole ring’s position

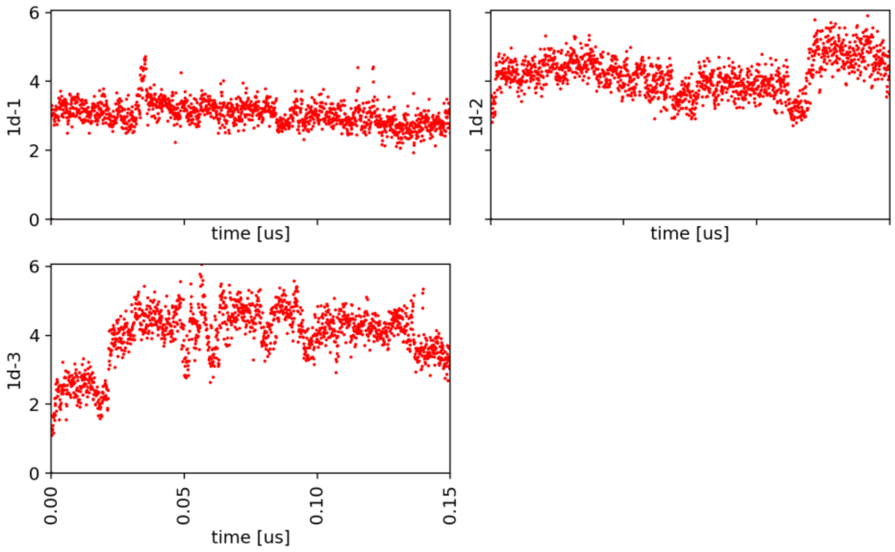

Figure SM-MOD-8. RMSD plot of the indole ring’s position in the simulations of 1d/hNK1R complexes.

RMSD is given in Angstrems.

DOTA fragment's position

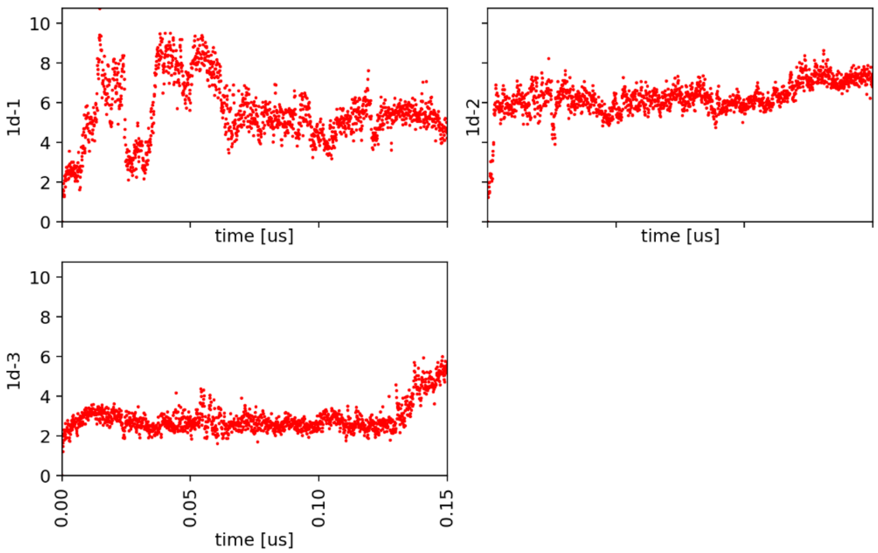

Figure SM-MOD-9. RMSD plot of the DOTA fragment's position in the simulations of 1d/hNK1R complexes.

RMSD is given in Angstrems.

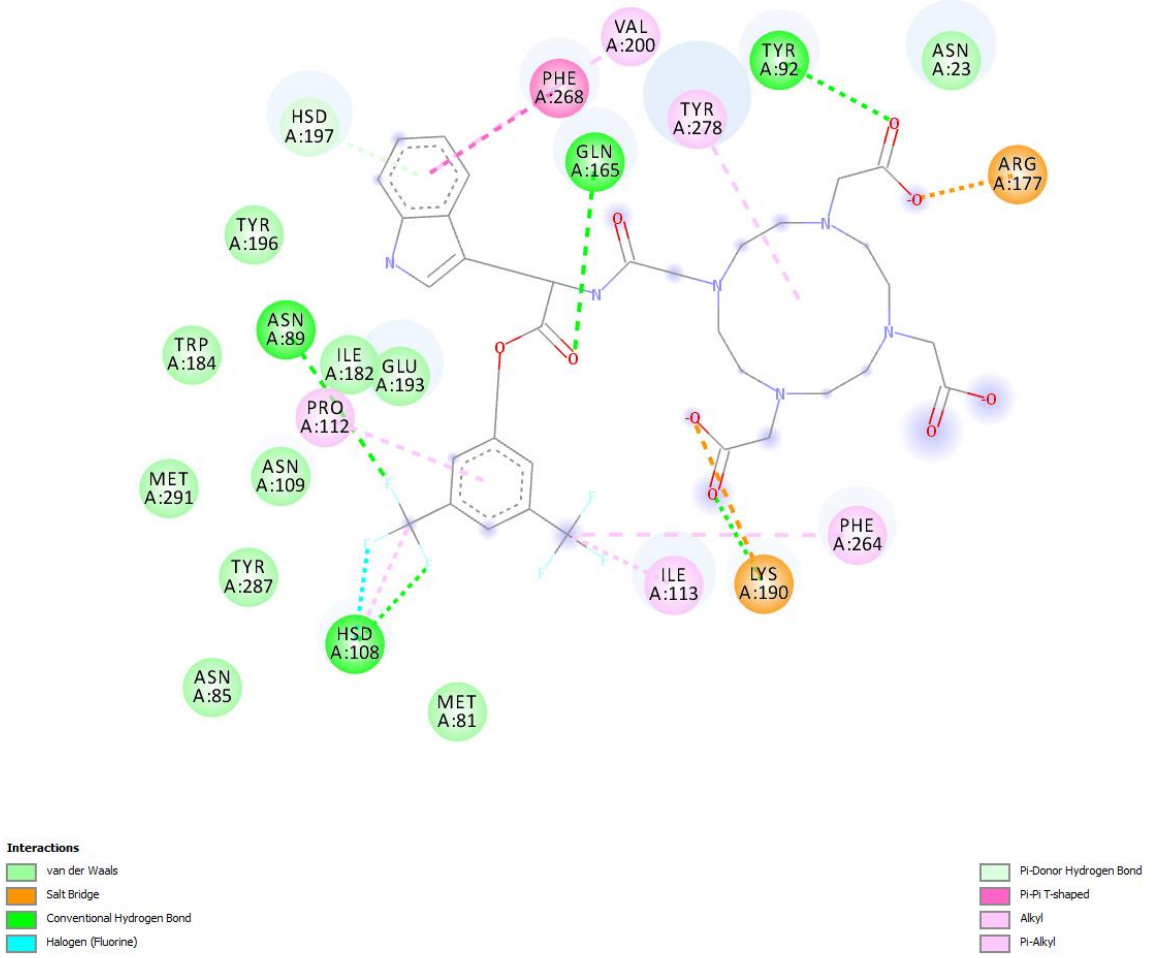

Figure SM-MOD-10. Scheme of the interactions of **1d** with the NK1R as found in the MD simulations (simulation **1d-1**, T = 150.0 ns). The interaction types are shown by colours as described in the legend.

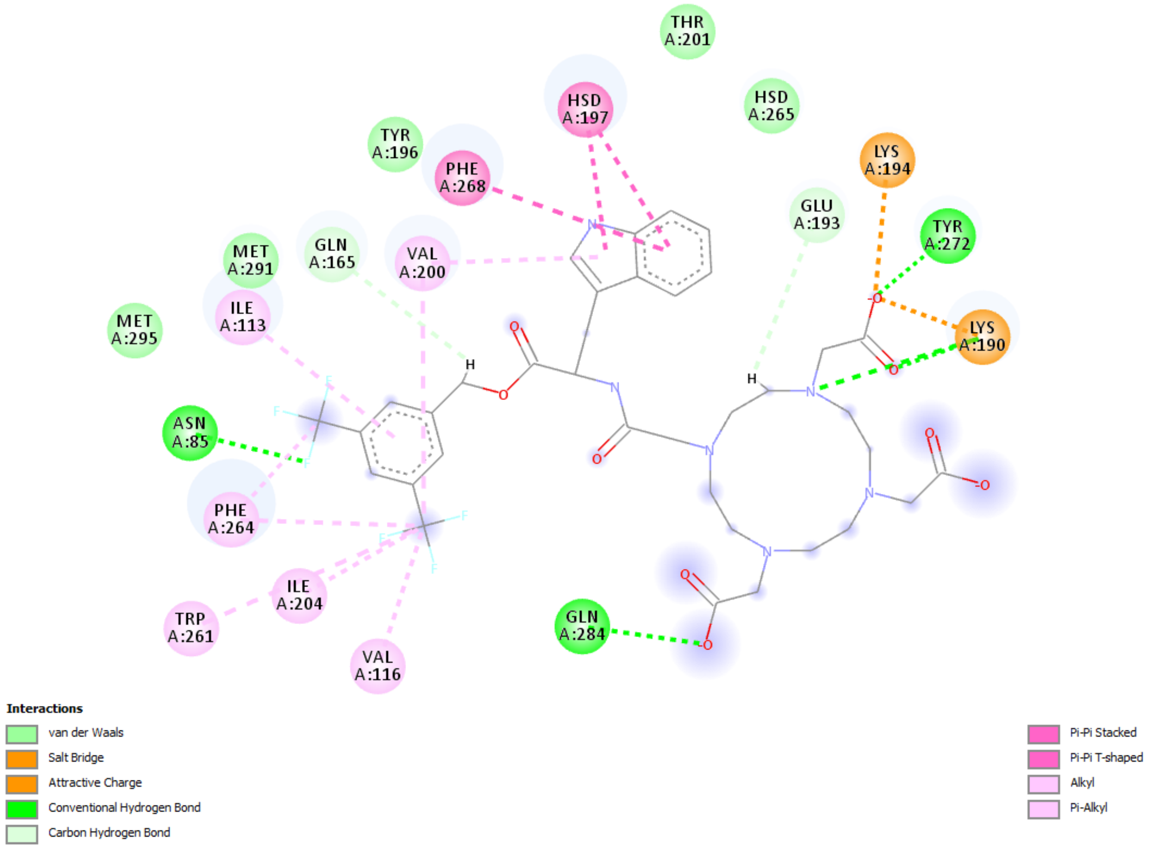

Figure SM-MOD-11. Scheme of the interactions of **1d** with the NK1R as found in the MD simulations (simulation **1d-2**, T = 150.0 ns). The interaction types are shown by colours as described in the legend.

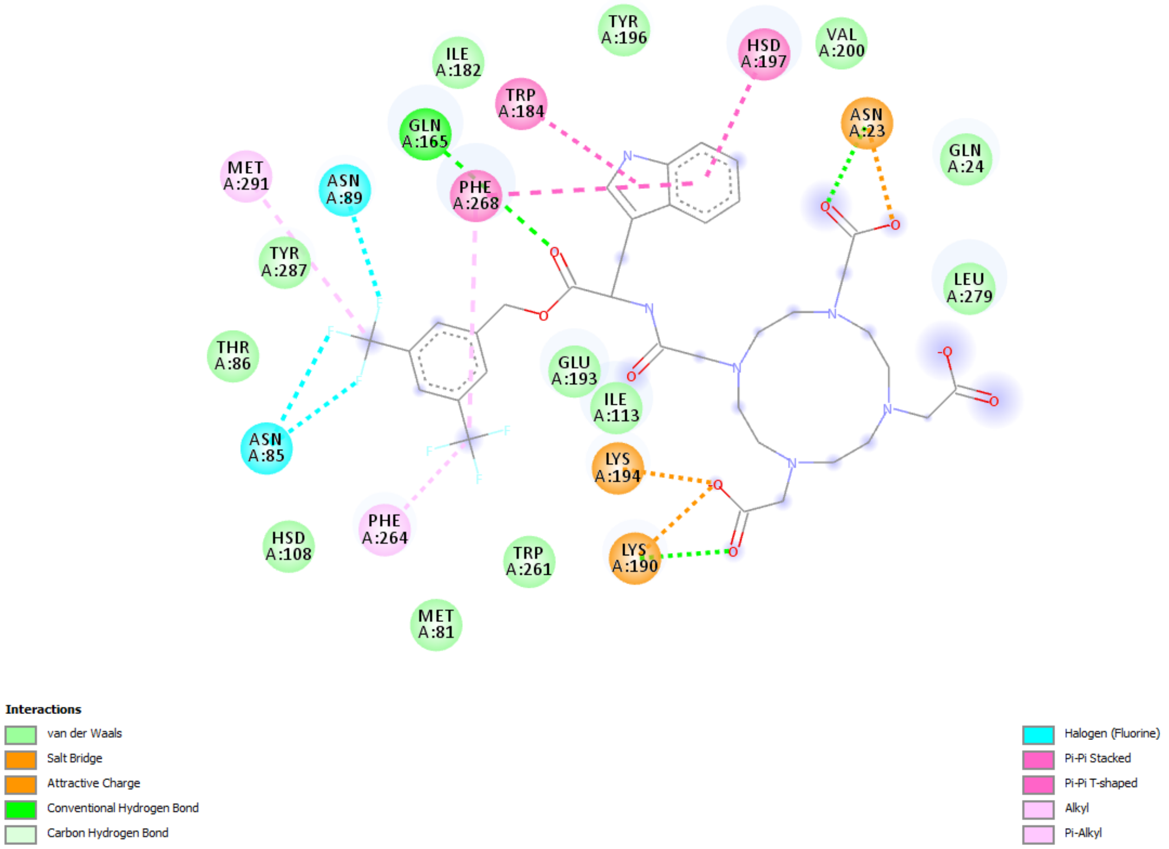

Figure SM-MOD-12. Scheme of the interactions of **1d** with the NK1R as found in the MD simulations (simulation **1d-3**, T = 150.0 ns). The interaction types are shown by colours as described in the legend.

Compound 5a

Ligand's position

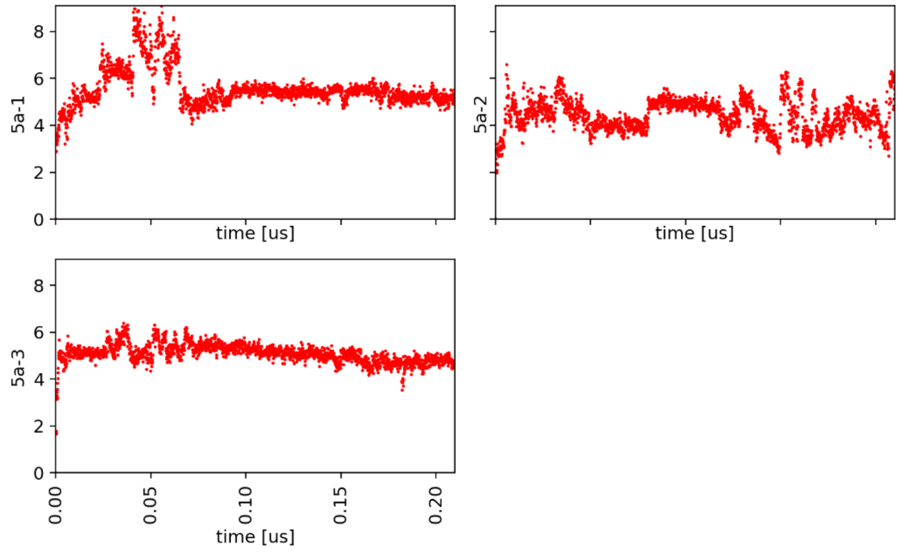

Figure SM-MOD-13. RMSD plot of the ligand position in the simulations of 5a/hNK1R complexes.

RMSD is given in Angstrems.

3,5-bistrifluoromethylbenzyl fragment's position

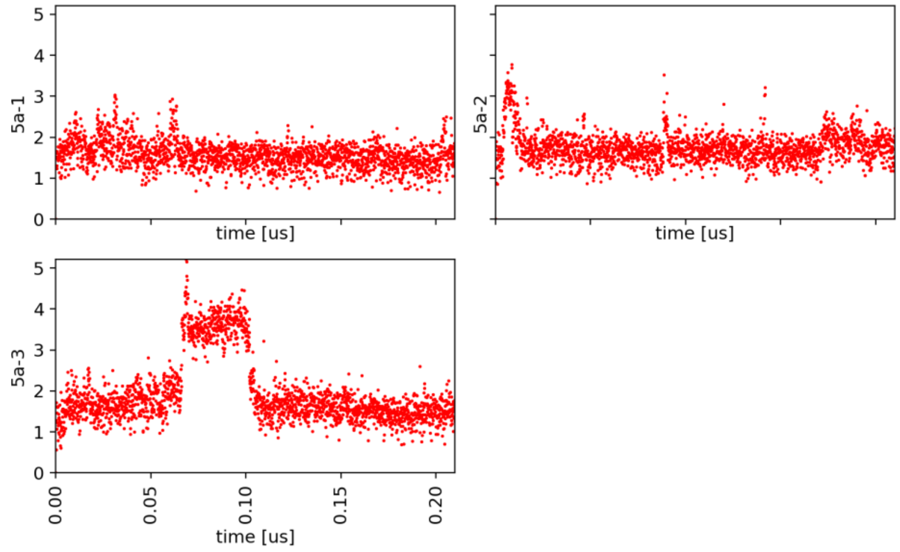

Figure SM-MOD-14. RMSD plot of the 3,5-bistrifluoromethylbenzyl fragment's position in the simulations of 5a/hNK1R complexes.

RMSD is given in Angstrems.

Indole ring's position

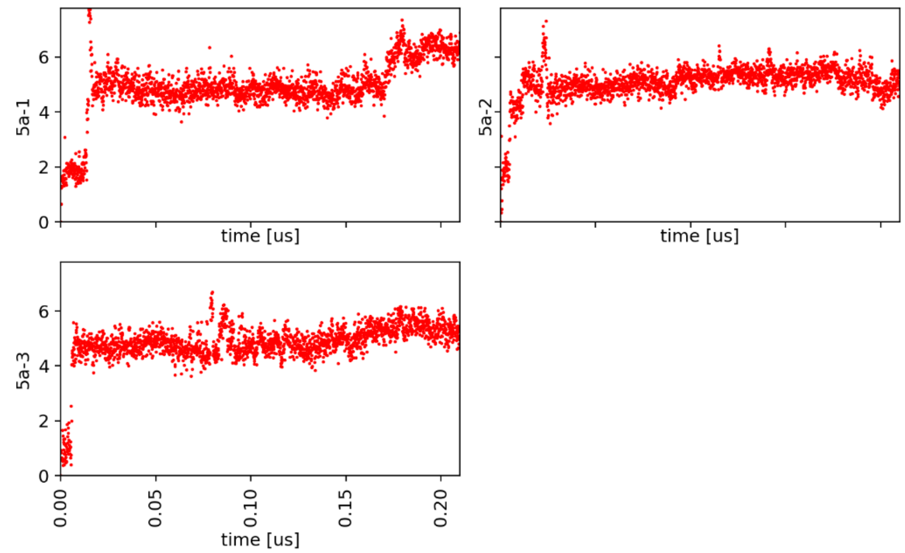

Figure SM-MOD-15. RMSD plot of the indole ring's position in the simulations of 5a/hNK1R complexes.

RMSD is given in Angstrems.

Boc-Ahx-Ahx- fragment's position

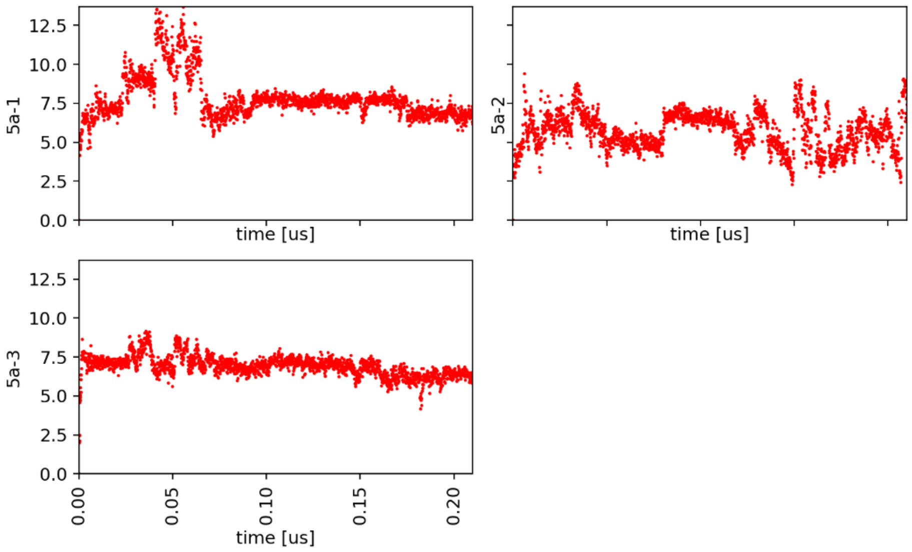

Figure SM-MOD-16. RMSD plot of the Boc-Ahx-Ahx fragment's position in the simulations of 5a/hNK1R complexes.

RMSD is given in Angstrems.

-Ahx1- (closer to Trp) fragment's position

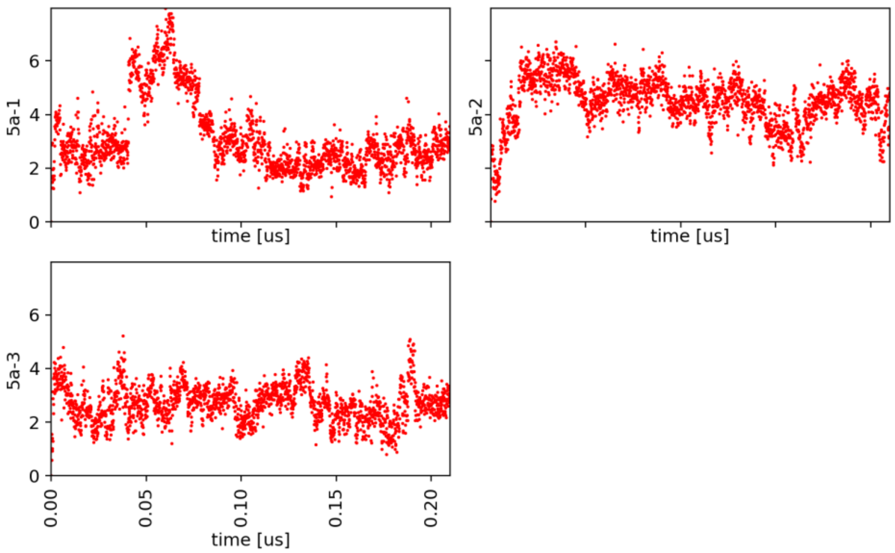

Figure SM-MOD-17. RMSD plot of the -Ahx1- (closer to Trp) fragment's position in the simulations of 5a/hNK1R complexes.

RMSD is given in Angstrems.

-Ahx2- (N-terminal) fragment's position

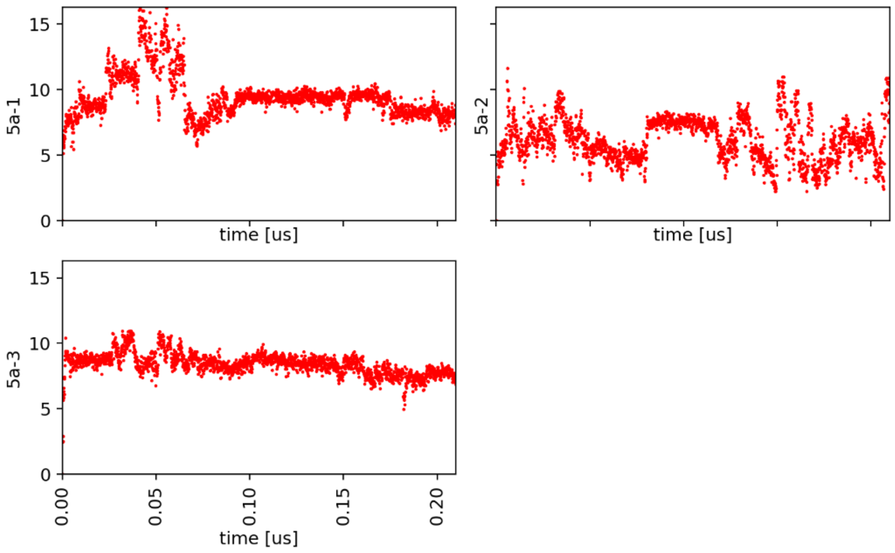

Figure SM-MOD-18. RMSD plot of the -Ahx2- (N-terminal) fragment's position in the simulations of 5a/hNK1R complexes.

RMSD is given in Angstrems.

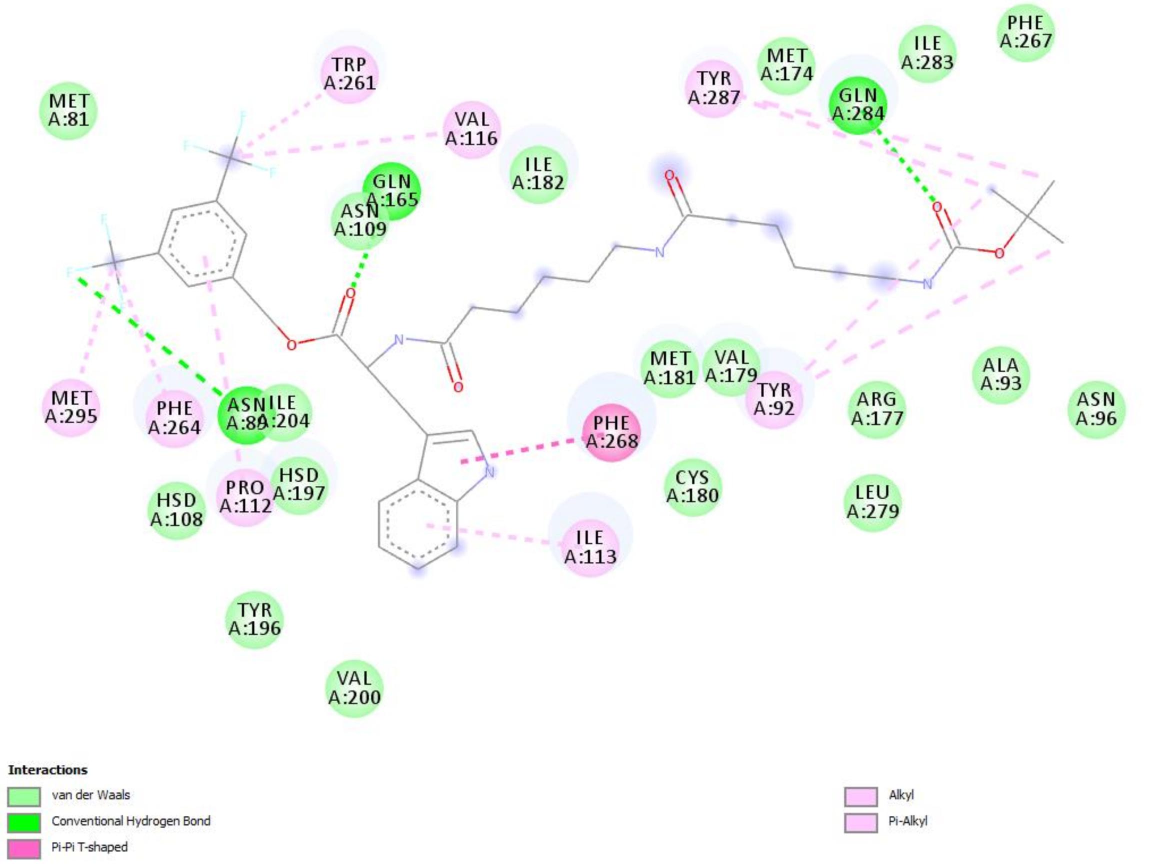

Figure SM-MOD-19. Scheme of the interactions of **5a** with the NK1R as found in the MD simulations (simulation **5a-1**, T = 210.0 ns). The interaction types are shown by colours as described in the legend.

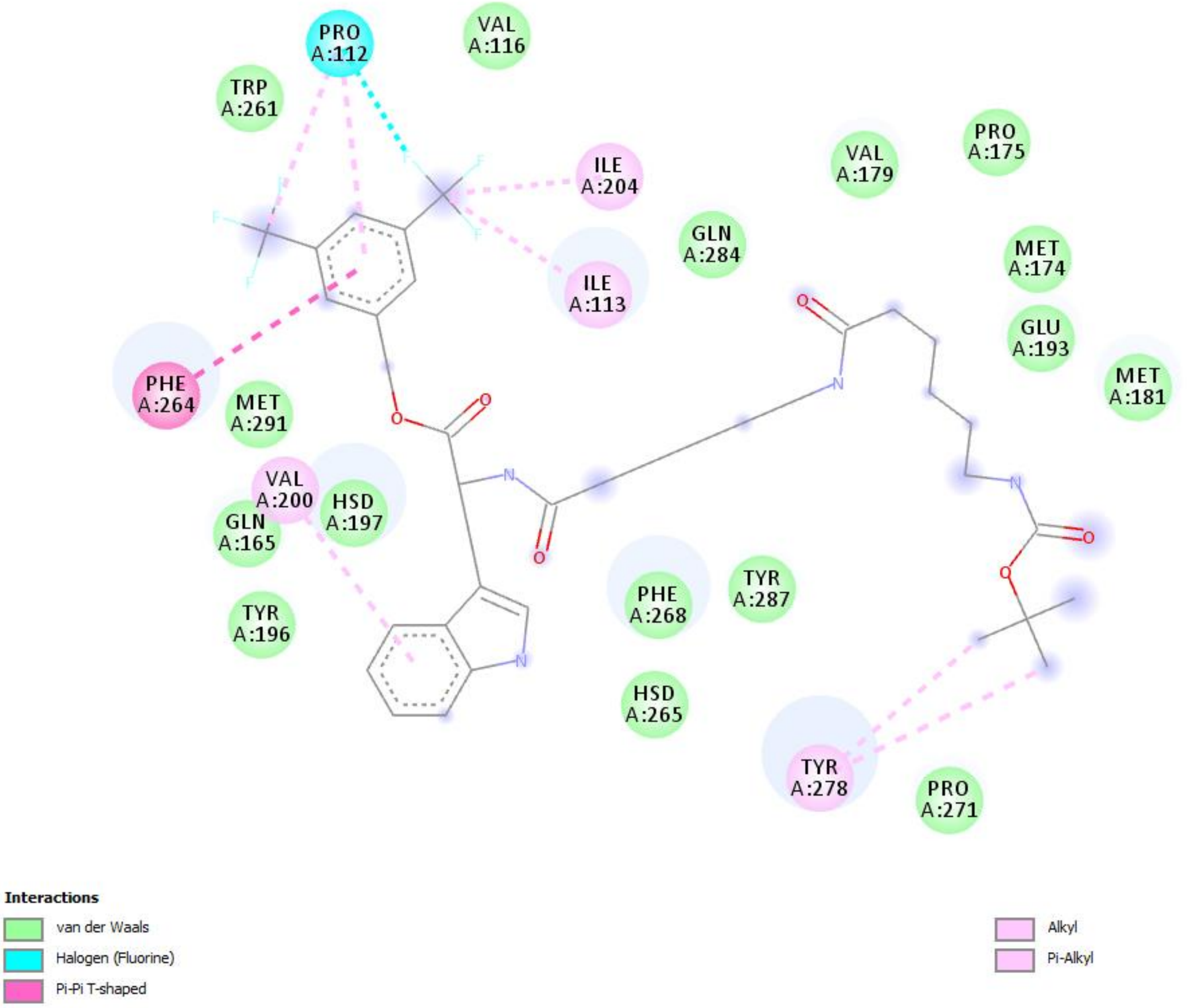

Figure SM-MOD-20. Scheme of the interactions of **5a** with the NK1R as found in the MD simulations (simulation **5a-2**, T = 210.0 ns). The interaction types are shown by colours as described in the legend.

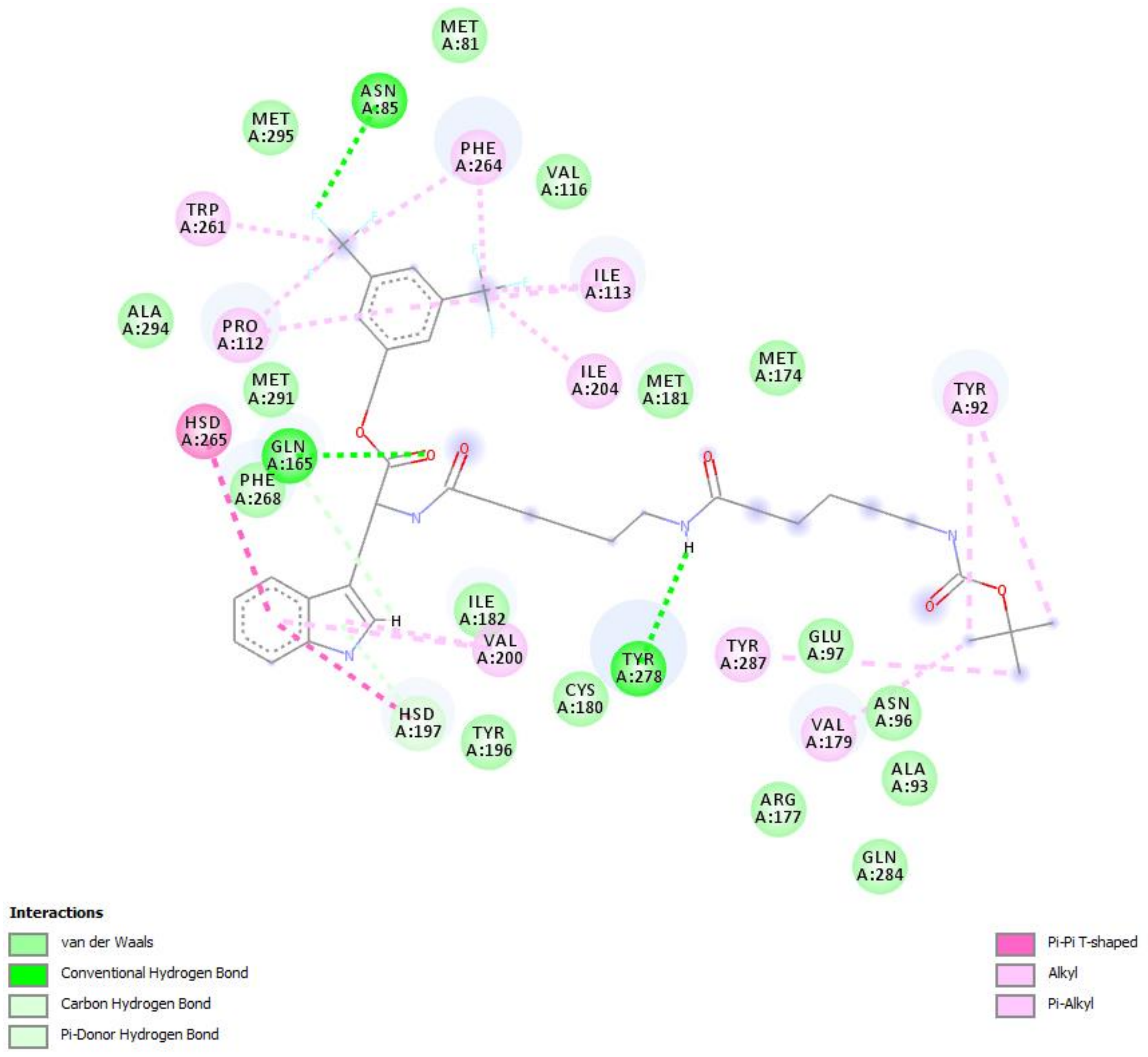

Figure SM-MOD-21. Scheme of the interactions of **5a** with the NK1R as found in the MD simulations (simulation **5a-3**, T = 210.0 ns). The interaction types are shown by colours as described in the legend.

Compound 5b

Ligand's position

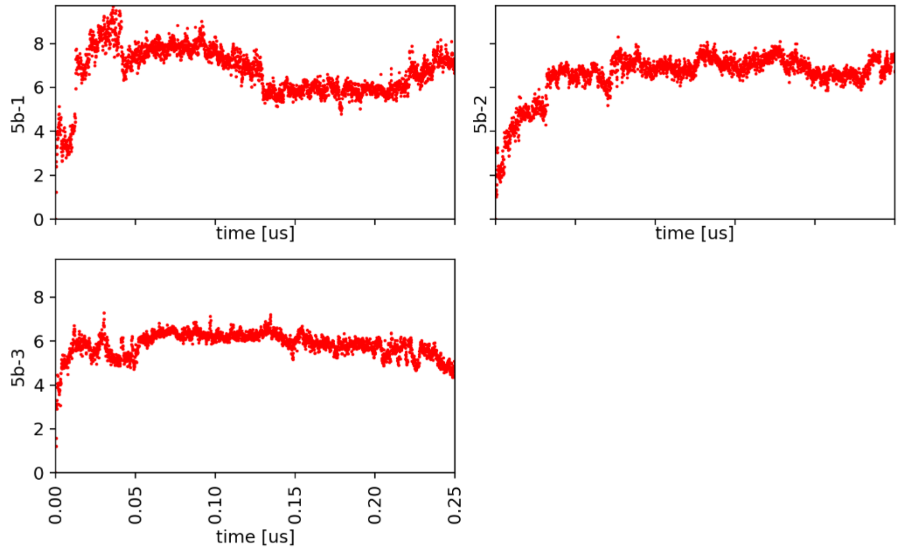

Figure SM-MOD-22. RMSD plot of the ligand position in the simulations of 5b/hNK1R complexes.

RMSD is given in Angstrems.

3,5-bistrifluoromethylbenzyl fragment's position

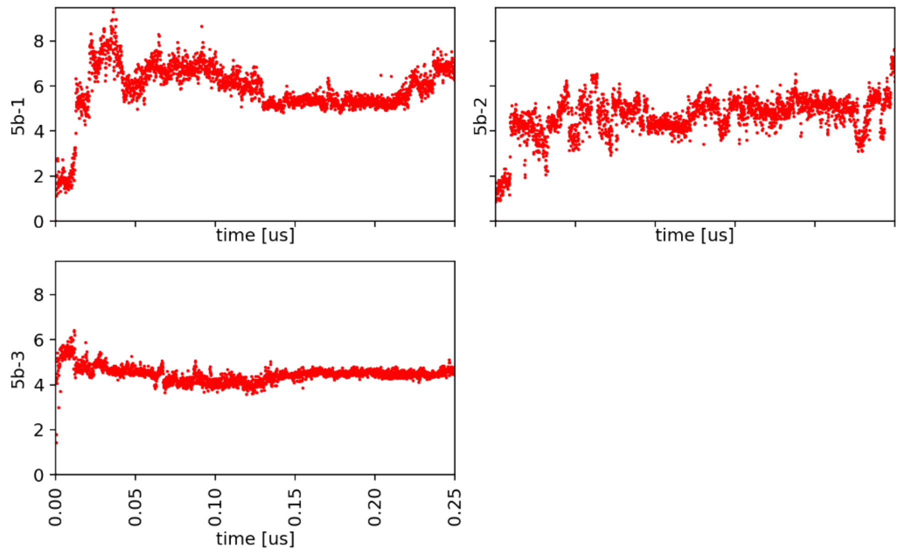

Figure SM-MOD-23. RMSD plot of the 3,5-bistrifluoromethylbenzyl fragment's position in the simulations of 5b/hNK1R complexes.

RMSD is given in Angstrems.

Indole ring’s position

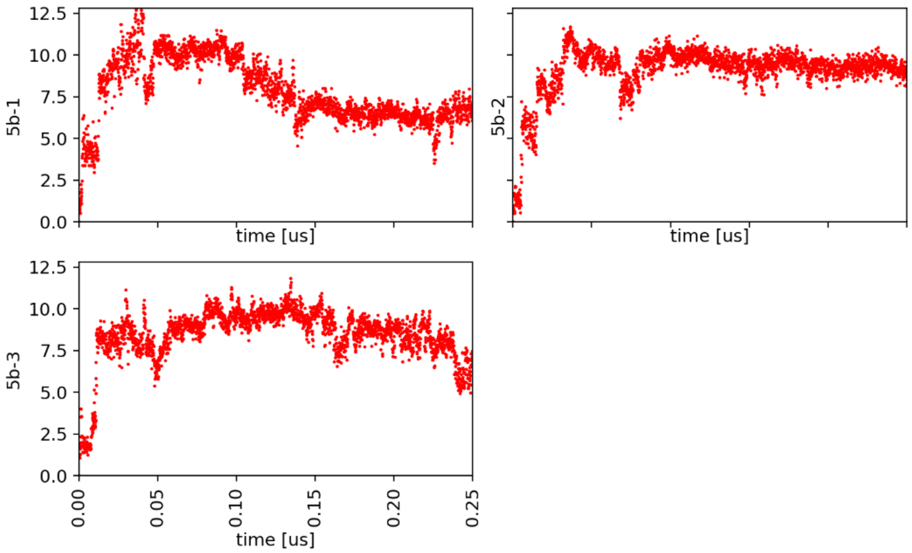

Figure SM-MOD-24. RMSD plot of the indole ring’s position in the simulations of 5b/hNK1R complexes.

RMSD is given in Angstrems.

NH_2-Ahx-Ahx- fragment’s position

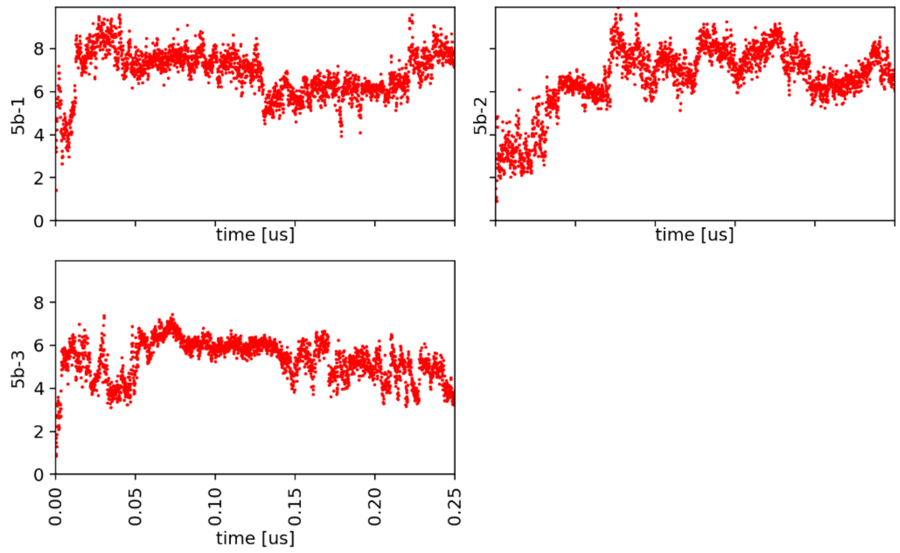

Figure SM-MOD-25. RMSD plot of the NH_2-Ahx-Ahx- fragment’s position in the simulations of 5b/hNK1R complexes.

RMSD is given in Angstrems.

-Ahx1- (closer to Trp) fragment's position

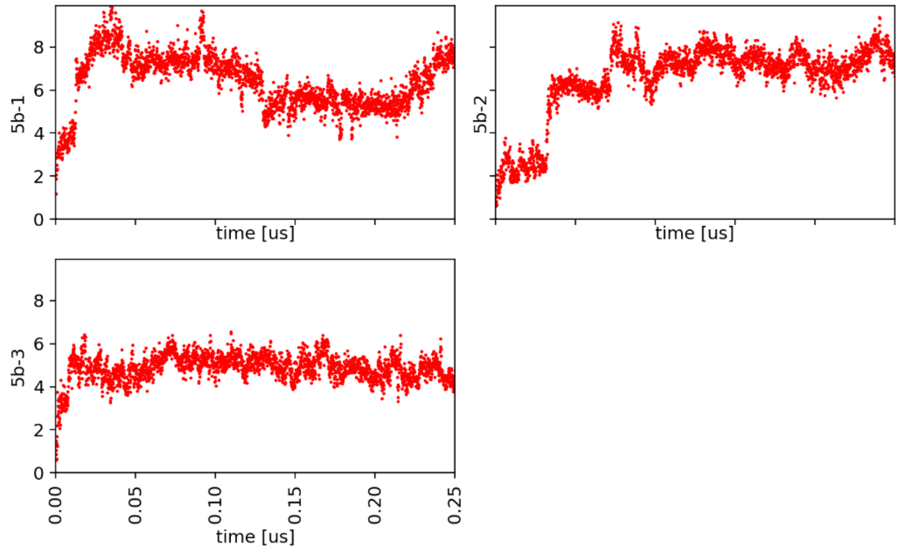

Figure SM-MOD-26. RMSD plot of the -Ahx1- (closer to Trp) fragment's position in the simulations of 5b/hNK1R complexes.

RMSD is given in Angstrems.

-Ahx2- (N-terminal) fragment's position

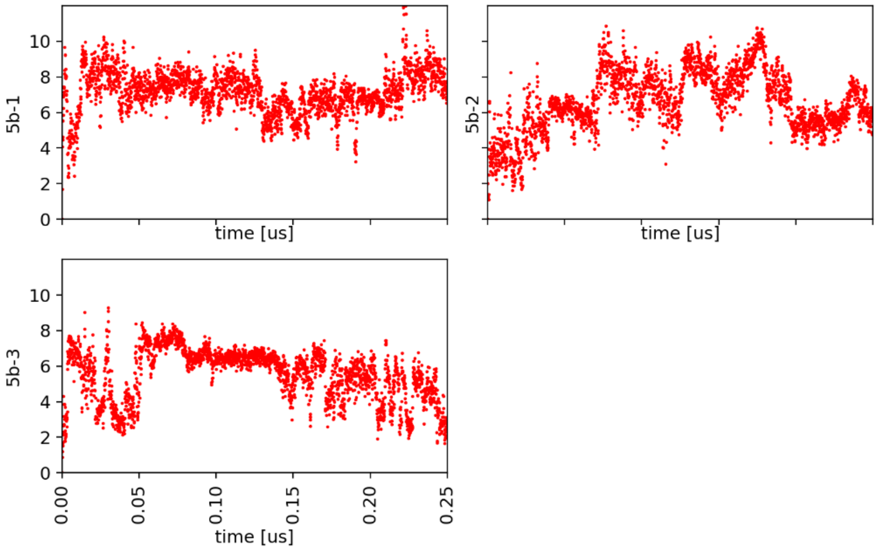

Figure SM-MOD-27. RMSD plot of the -Ahx2- (N-terminal) fragment's position in the simulations of 5b/hNK1R complexes.

RMSD is given in Angstrems.

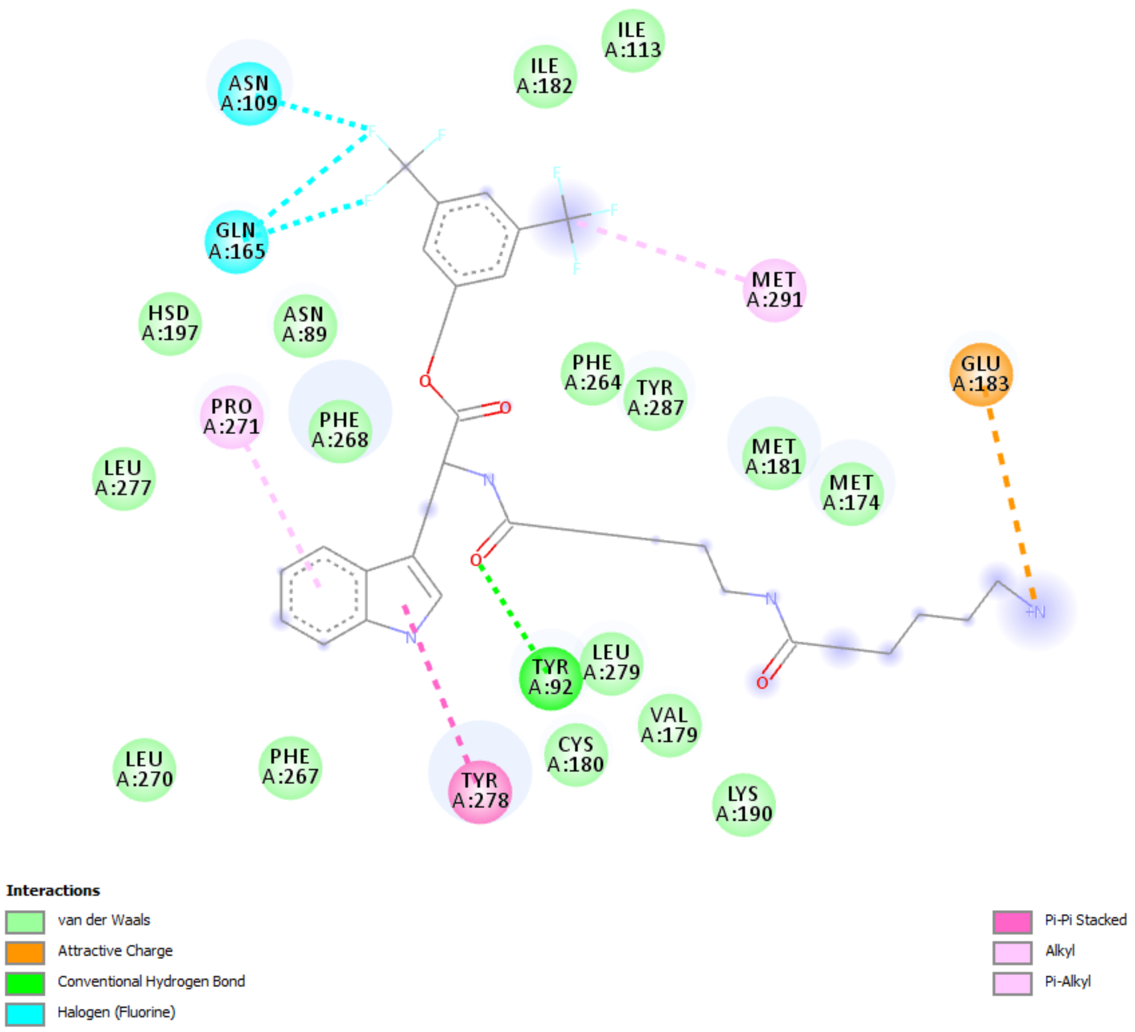

Figure SM-MOD-28. Scheme of the interactions of **5b** with the NK1R as found in the MD simulations (simulation **5b-1**, T = 250.0 ns). The interaction types are shown by colours as described in the legend.

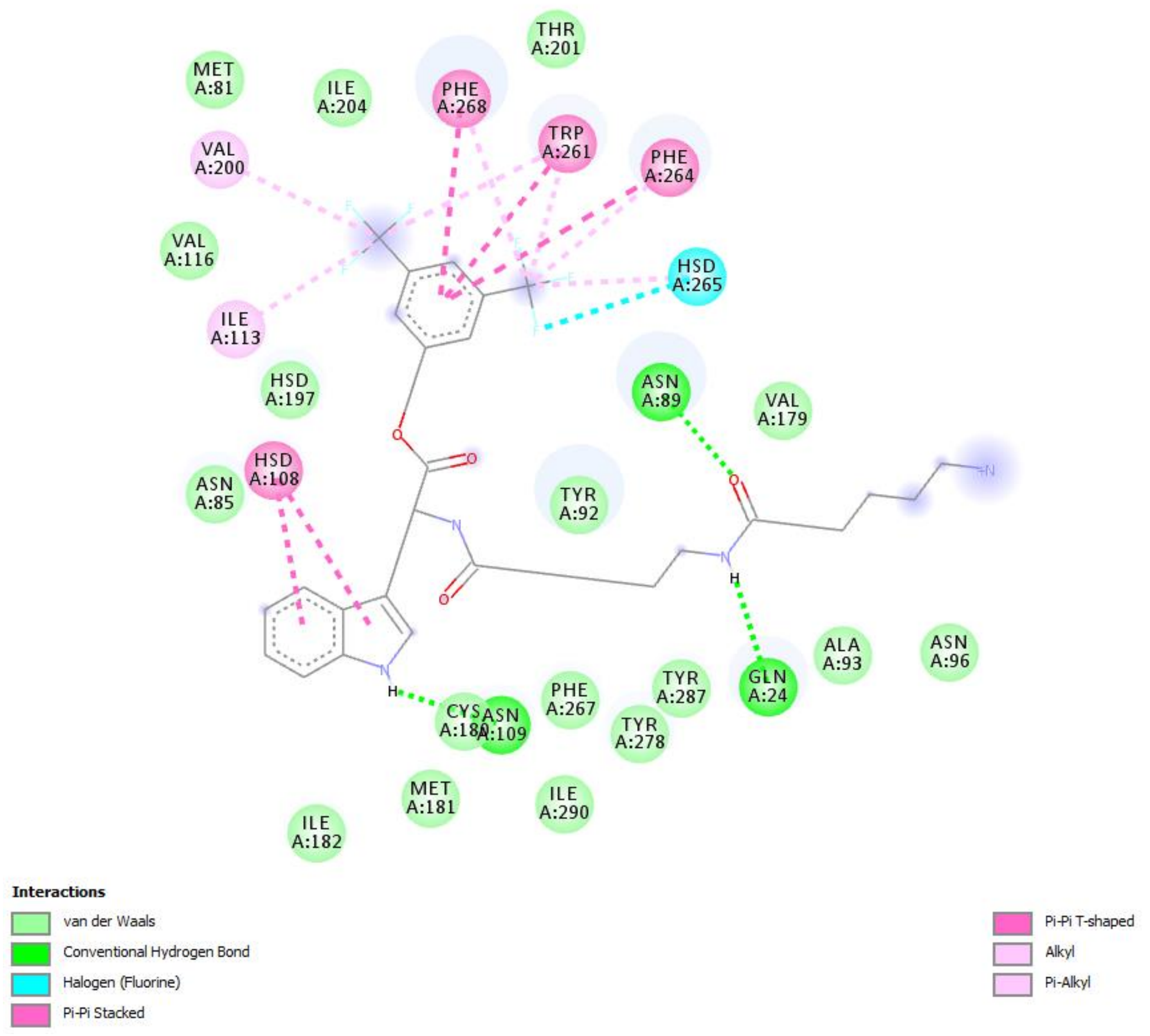

Figure SM-MOD-29. Scheme of the interactions of **5b** with the NK1R as found in the MD simulations (simulation **5b-2**, T = 250.0 ns). The interaction types are shown by colours as described in the legend.

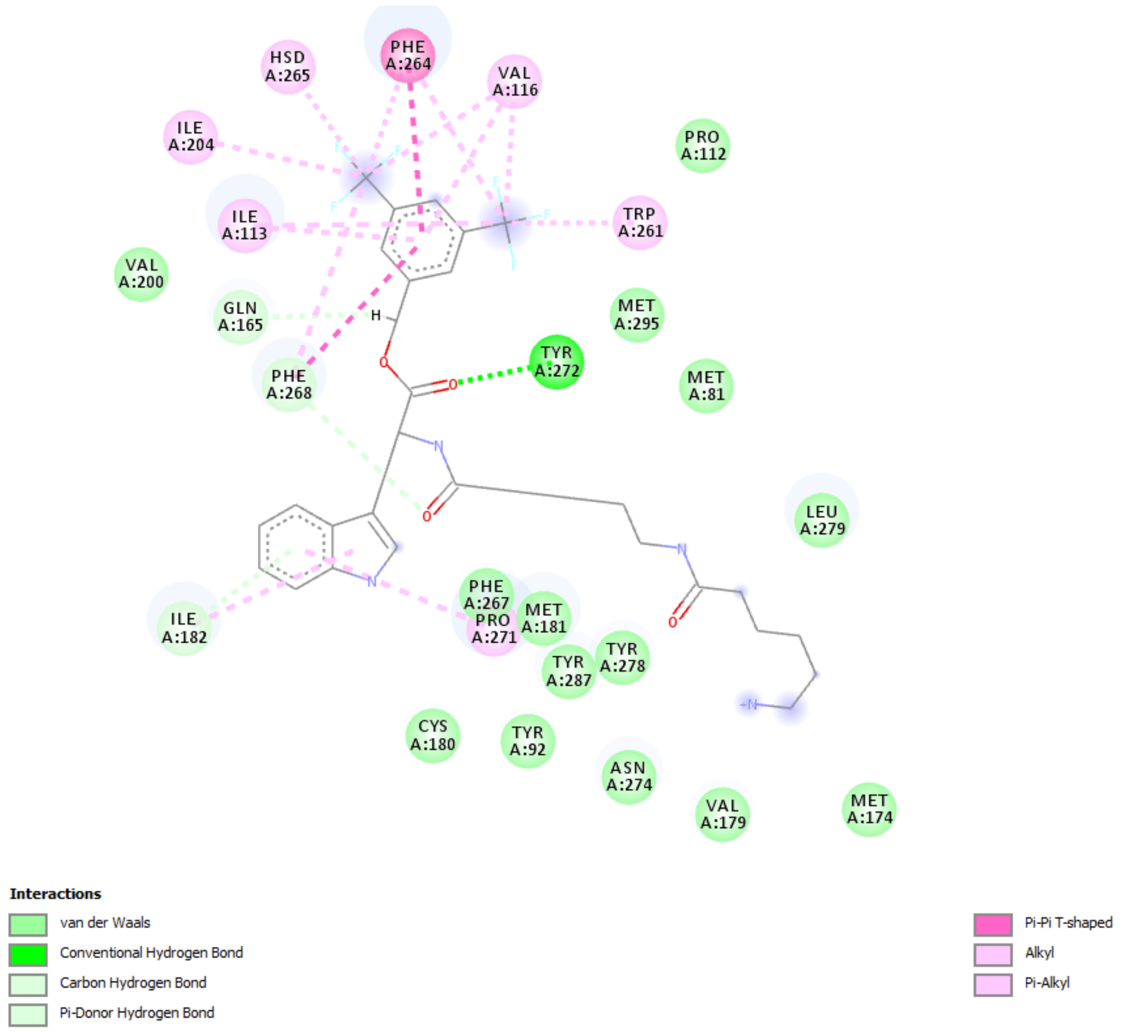

Figure SM-MOD-30. Scheme of the interactions of **5b** with the NK1R as found in the MD simulations (simulation **5b-3**, T = 250.0 ns). The interaction types are shown by colours as described in the legend.

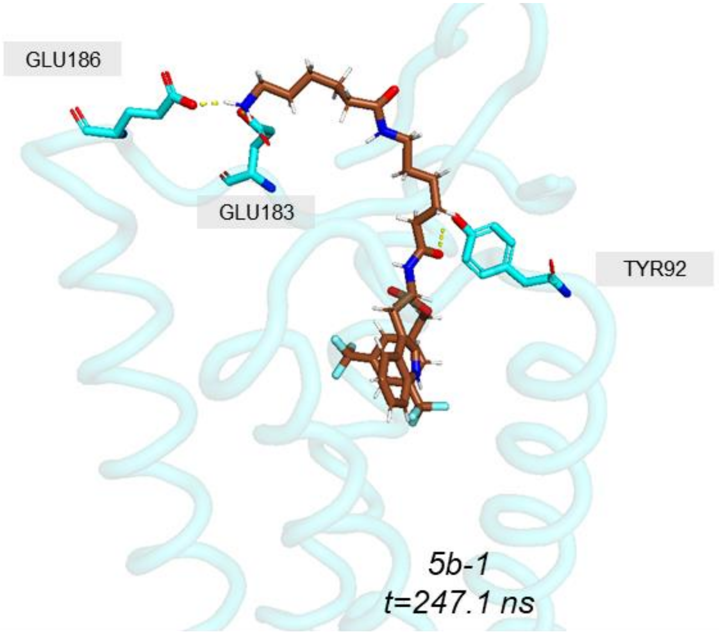

Figure SM-MOD-31. Compound **5b** (brown) in the hNK1R binding pocket. A snapshot from 5b-1 MD simulation (t=247.1 ns). The receptor (light blue) displayed in a simplified manner (only several helices as ribbons and only several interacting residues as sticks).

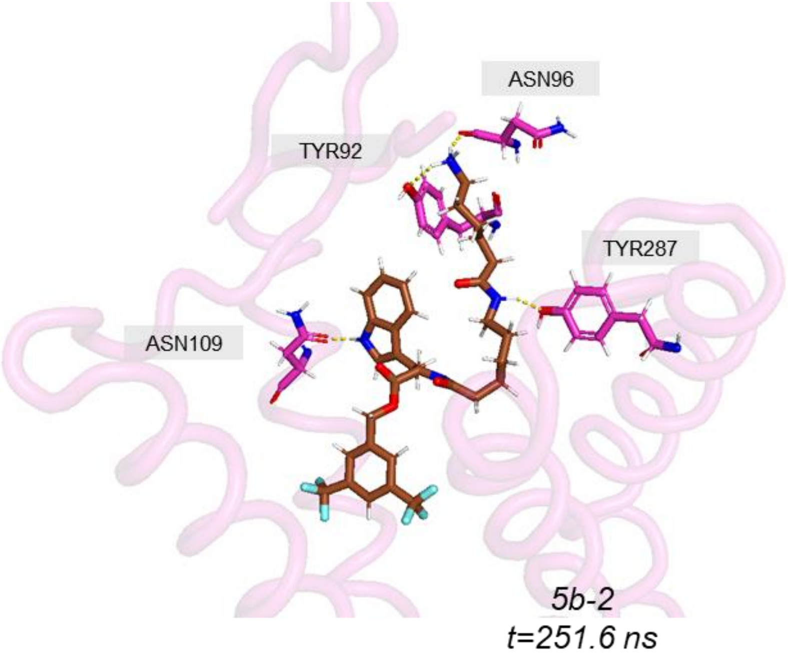

Figure SM-MOD-32. Compound **5b** (brown) in the hNK1R binding pocket. A snapshot from 5b-2 MD simulation (t=251.6 ns). The receptor (pink) displayed in a simplified manner (only several helices as ribbons and only several interacting residues as sticks).

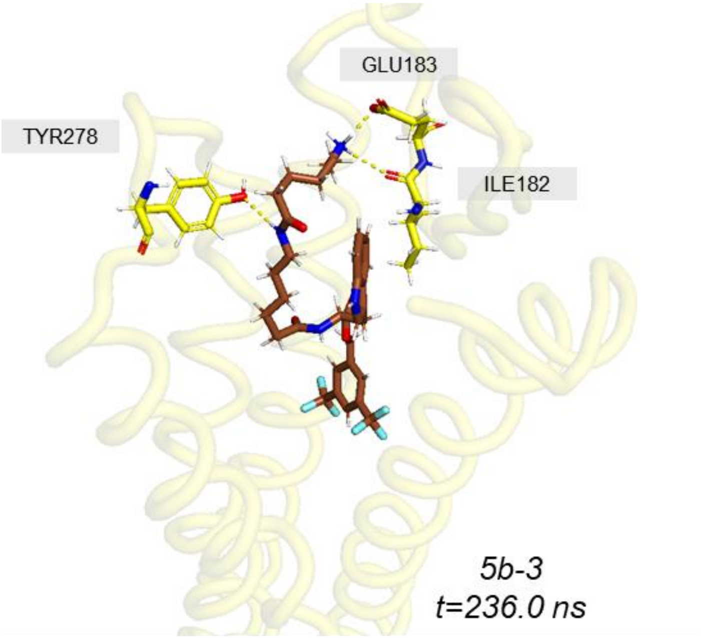

Figure SM-MOD-33. Compound **5b** (brown) in the hNK1R binding pocket. A snapshot from 5b-3 MD simulation (t=236.0 ns). The receptor (yellow) displayed in a simplified manner (only several helices as ribbons and only several interacting residues as sticks).
